# Comprehensive analysis of nuclear export of herpes simplex virus type 1 tegument proteins and their Epstein‐Barr virus orthologs

**DOI:** 10.1111/tra.12627

**Published:** 2019-01-11

**Authors:** Christina Funk, Verena Raschbichler, Diana Lieber, Jens Wetschky, Eileen K. Arnold, Jacqueline Leimser, Michael Biggel, Caroline C. Friedel, Zsolt Ruzsics, Susanne M. Bailer

**Affiliations:** ^1^ Fraunhofer Institute for Interfacial Engineering and Biotechnology IGB Stuttgart Germany; ^2^ Max von Pettenkofer‐Institute Ludwig‐Maximilians‐University Munich Munich Germany; ^3^ Institute of Virology Ulm University Medical Center Ulm Germany; ^4^ Institute of Interfacial Process Engineering and Plasma Technology University of Stuttgart Stuttgart Germany; ^5^ Institute for Informatics Ludwig‐Maximilians‐University Munich Munich Germany; ^6^ Institute of Virology, Medical Center—University of Freiburg, Medical Faculty University of Freiburg Freiburg Germany

**Keywords:** CRM1, EBV, herpesviruses, HSV1, NEX‐TRAP, nuclear export, tegument proteins

## Abstract

Morphogenesis of herpesviral virions is initiated in the nucleus but completed in the cytoplasm. Mature virions contain more than 25 tegument proteins many of which perform both nuclear and cytoplasmic functions suggesting they shuttle between these compartments. While nuclear import of herpesviral proteins was shown to be crucial for viral propagation, active nuclear export and its functional impact are still poorly understood. To systematically analyze nuclear export of tegument proteins present in virions of Herpes simplex virus type 1 (HSV1) and Epstein‐Barr virus (EBV), the Nuclear EXport Trapped by RAPamycin (NEX‐TRAP) was applied. Nine of the 22 investigated HSV1 tegument proteins including pUL4, pUL7, pUL11, pUL13, pUL21, pUL37d11, pUL47, pUL48 and pUS2 as well as 2 out of 6 EBV orthologs harbor nuclear export activity. A functional leucine‐rich nuclear export sequence (NES) recognized by the export factor CRM1/Xpo1 was identified in six of them. The comparison between experimental and bioinformatic data indicates that experimental validation of predicted NESs is required. Mutational analysis of the pUL48/VP16 NES revealed its importance for herpesviral propagation. Together our data suggest that nuclear export is an important feature of the herpesviral life cycle required to co‐ordinate nuclear and cytoplasmic processes.

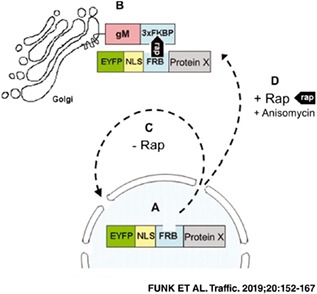

## 1 INTRODUCTION

The family of herpesviruses can be divided into the three subfamilies alpha‐, beta‐ and gamma‐herpesviruses based on characteristics such as cell tropism, pathogenicity and the site of latency. Herpes simplex virus type 1 (HSV1), a member of the alpha‐herpesvirus subfamily, causes recurrent facial lesions or encephalitis. Epstein‐Barr virus (EBV) that causes infectious mononucleosis and diverse lymphoproliferative disorders is a member of the gamma‐herpesviruses.

Morphogenesis of herpesviral virions is initiated in the host nucleus by packaging of the viral genomes into capsids but is completed at cytoplasmic membranes with the secondary envelopment of the capsids. Nuclear capsids seem to associate with several partially characterized tegument proteins,^1^, ^2^ final tegumentation, however, occurs during secondary envelopment. HSV1 virions released to the extracellular milieu contain more than 25 tegument proteins^1^ (Table 1). These are expected to perform dual roles during herpesviral infection, structural ones in the viral particle and regulatory ones by modulating viral and/or cellular functions. This includes several well‐known transcriptionally active proteins released by the incoming virion. A prominent example is pUL48/VP16 that activates immediate early genes.^3^ ICP0, another tegument protein that performs various functions during the herpesviral life cycle, is transcriptionally active during the immediate early, early and late stage of viral replication (Reference 4 and references therein). Comprehensive analysis revealed the active nuclear import of many tegument proteins independent of other viral partners^5^, ^6^ (our unpublished data). Active export of viral proteins from the nucleus to the cytoplasm, however, has in general received little attention. HSV tegument proteins proposed to shuttle include pUL37/ICP32,^7^ VP13/14/pUL47 (8 and references therein), ICP34.5/RL1,^9^ pUL41/vhs^10^) and pUL4,^11^, ^12^ even less is known about nuclear export of EBV proteins. Overall, the functional relevance of nucleo‐cytoplasmic shuttling of individual herpesviral proteins remains to be explored.

**Table 1:** HSV1 tegument proteins present in mature virions^1^ and their homologs

HSV1 tegument	Other name/function	MW HSV1^a^	VZV	MW VZV^b^	HCMV	MW HCMV^c^	EBV	MW EBV^d^
RL1	ICP34.5	26.2	‐		‐		‐	
RL2	ICP0	78.5	ORF61	50.9	‐		‐	
pUL4		21.5	ORF56	27.2	‐		‐	
pUL7		33.1	ORF53	37.4	UL103	28.6	pBBRF2	31.3
pUL11		10.5	ORF49	8.9	UL99	20.9	pBBLF1	8.5
pUL13		57.2	ORF47	57.4	UL97	78.3	pBGLF4	48.4
pUL14		23.9	ORF46	22.5	UL95	57.3	pBGLF3	37.7
pUL16		40.4	ORF44	40.2	UL94	38.3	pBGLF2	36.9
pUL21		57.6	ORF38	60.4	UL87	105.0	pBTRF1	44.3
pUL23	TK	41.0	ORF36	37.8	‐		pBXLF2	78.3
pUL36	Large tegument	335.9	ORF22	306.3	UL48	253.2	pBPLF1	338.0
pUL37	ICP32	120.6	ORF21	115.8	UL47	110.0	pBOLF1	132.7
pUL41	Vhs	54.9	ORF17	51.4	‐		‐	
pUL46	VP11/12	78.2	ORF12	74.3	‐		‐	
pUL47	VP13/14	73.8	ORF11	91.8	‐		‐	
pUL48	VP16/ICP25	54.3	ORF10	46.6	‐		‐	
pUL49	VP22	32.3	ORF9	32.8	‐		‐	
pUL50	dUTPase	39.1	ORF8	44.8	‐		pBLLF3	31.0
pUL51		25.5	ORF7	28.2	UL71	39.9	pBSRF1	23.9
pUL55		20.5	ORF33	66.0	‐		‐	
RS1	ICP4	132.8	ORF62/71	140.0	‐		‐	
pUS2		32.5	‐		‐		‐	
pUS3		52.8	ORF66	43.7	‐		‐	
pUS10		34.1	ORF64/69	19.9	‐		‐	
pUS11		17.8	‐		‐		‐	

The RL1, RL2, and RS1 tegument proteins could not be analyzed since their coding sequence was unavailable.

a https://www.uniprot.org/uniprot/?query=proteome:UP000009294 Human herpesvirus 1 (strain 17) (HHV‐1).

b https://www.uniprot.org/uniprot/?query=proteome:UP000002602 Varicella‐zoster virus (strain Dumas) (HHV‐3) (Human herpesvirus 3).

c https://www.uniprot.org/uniprot/?query=proteome:UP000000938 Human cytomegalovirus (strain Merlin) (HHV‐5) (Human herpesvirus 5).

d https://www.uniprot.org/uniprot/?query=proteome:UP000153037 Epstein‐Barr virus (strain B95‐8) (HHV‐4) (Human herpesvirus 4).

Selective and active nucleo‐cytoplasmic exchange of proteins is mediated by soluble transport receptors of the importin β‐family (also called importins/exportins or karyopherins;^13^). Several protein sequences have been identified that mediate recognition of the cargo by transport factors. Nuclear import is often mediated by nuclear import signals including the “classical” SV40 large tumor antigen (SV40TAg)‐like nuclear localization signal (NLS), and the bipartite NLS consisting of two stretches of basic residues.^14^ Nuclear export of proteins is primarily mediated by a leucine‐rich nuclear export signal (NES). Consensus sequences have been deduced for basic mono‐ and bipartite NLSs recognized by the importin‐α/β heterodimer,^14^ for signals recognized by four other import factors of the importin β family^15^ and for leucine‐rich NESs.^16^, ^17^, ^18^ While NLSs with basic stretches can be identified with high confidence based on consensus sequences, prediction of a functional NES using bioinformatic tools is difficult and often results in false positives and false negatives.^19^, ^20^, ^21^, ^22^, ^23^ Therefore, the export activity of a protein needs to be determined experimentally, in vivo and in the context of the folded protein, and predicted NESs require experimental confirmation.

To analyze nuclear export of proteins, we previously established the NEX‐TRAP assay (Nuclear EXport Trapped by RAPamycin^12^). Here, a protein X to be tested for nuclear export is fused to EYFP for visualization, to the classical SV40 NLS for constitutive nuclear import and to the FK506‐Rapamycin (FR)‐binding domain (FRB) for dimerization. The EYFP‐NLS‐FRB‐Protein X in general exceeds the diffusion limit of the nuclear pore disabling its passive export.^24^ The reporter protein gM‐FKBP is based on a fusion of HSV1 glycoprotein M (gM), a resident integral membrane protein of the *trans‐*Golgi network (TGN^25^), and three tandem repeats of the immunophilin FK506‐binding protein‐12 (FKBP) exposed to the cytoplasm. Upon co‐expression of both proteins, gM‐FKBP locates to the TGN while an EYFP‐NLS‐FRB‐Protein X with nuclear export activity continuously shuttles between nucleus and cytoplasm but accumulates in the nucleus at steady‐state. In presence of Rapamycin, dimerization of the FRB and FKBP domains occurs thereby trapping a shuttling EYFP‐NLS‐FRB‐Protein X at the reporter protein gM‐FKBP which results in a permanent and concentrated signal at the TGN.

A systematic analysis was performed to determine the potential nuclear export activity of HSV1 and EBV tegument proteins. NEX‐TRAP analysis showed that 9 of the 22 investigated HSV1 tegument proteins harbor nuclear export activity. Mutagenesis identified a leucine‐rich NES suggested by bioinformatic prediction in six of these tegument proteins, CRM1/Xpo1‐dependent export of most of them was shown upon addition of Leptomycin B. Further analysis identified actively exported tegument proteins of the gamma‐herpesvirus EBV that in part are conserved throughout the human herpesvirus family. Functional analysis of pUL48/VP16 demonstrated that its nuclear export is important for herpesviral propagation. Together our data suggest that nuclear export is an essential feature of the herpesviral life cycle potentially coordinating nuclear and cytoplasmic processes.

## 2 RESULTS

### 2.1 Comprehensive analysis of nuclear export of HSV1 tegument proteins

**Figure 1:**
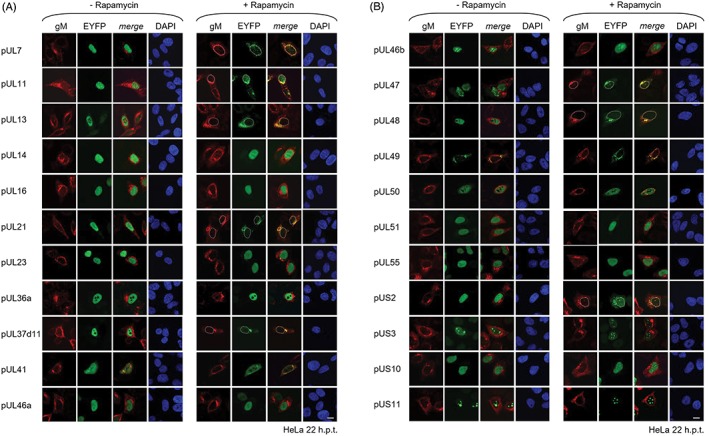
Comprehensive analysis of nuclear export activity of Herpes simplex virus type 1 (HSV1) tegument proteins. To determine the nuclear export activity of 22 HSV1 tegument proteins,^1^ the Nuclear EXport Trapped by RAPamycin (NEX‐TRAP) assay was performed. HeLa cells were transiently co‐transfected with the plasmid encoding gM‐FKBP and one plasmid of a collection of EYFP‐NLS‐FRB‐tagged tegument proteins. Following incubation of the cells with anisomycin and rapamycin, cells were analyzed by indirect immunofluorescence using rabbit anti‐gM antibodies followed by secondary reagents. EYFP‐tagged proteins were visualized directly. Nuclei were visualized by Dapi staining. The bar corresponds to 10 μm. Nuclei are marked by dashed lines

To comprehensively analyze the nuclear export activity of the HSV1 tegument proteins, the previously established NEX‐TRAP assay was applied.^12^ With exception of RL1, RL2 and RS1, all tegument proteins could be cloned as N‐terminal fusions with EYFP‐NLS‐FRB and were transiently co‐expressed with the reporter protein gM‐FKBP in HeLa cells. All proteins were expressed in full length except for pUL36a (aa 1‐1000), pUL36b (aa 1001‐2000), UL37d11 (aa 12‐1124), UL46a (aa 1‐336) and UL46b (aa 337‐719). Twenty hours following transfection of the dual vector system encoding these proteins, the transfected cells were either incubated with Rapamycin or left untreated. In absence of Rapamycin, most EYFP‐NLS‐FRB fusion proteins were located to the nucleus while gM‐FKBP was accumulated at the TGN (Figure 1, left panel). pUL41, pUL49 and pUL50 were located both in the cytoplasm and nucleus disabling their analysis by NEX‐TRAP. Upon addition of Rapamycin, pUL4,^12^ pUL7, pUL11, pUL13, pUL21, pUL37d11, pUL47, pUL48 and pUS2 were depleted from the nucleus and co‐localized with gM‐FKBP at the TGN consistent with their active nuclear export (Figure 1, right panel). In contrast, all other tegument proteins remained in the nucleus (Figure 1, right panel). Thus, a group of nine HSV1 tegument proteins including pUL4,^12^ pUL7, pUL11, pUL13, pUL21, pUL37d11, pUL47, pUL48 and pUS2 exhibited nuclear export activity.

### 2.2 Validation of the nuclear export activity of HSV1 tegument proteins

**Figure 2:**
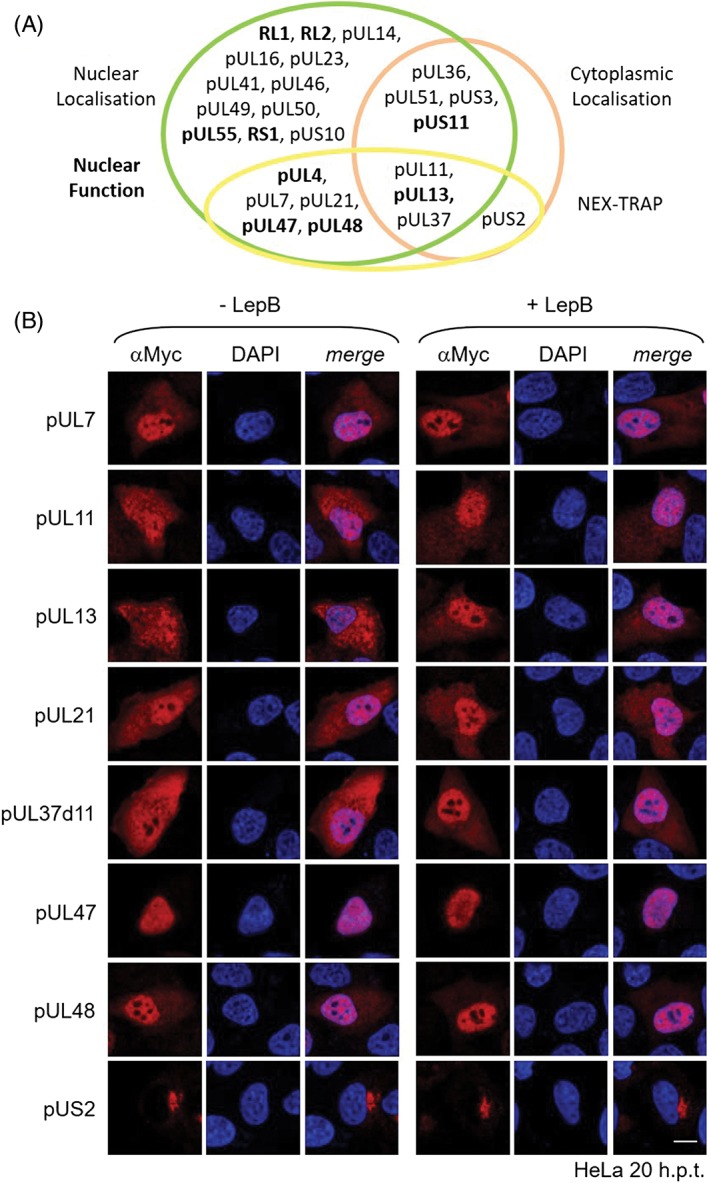
Validation of nuclear export activity of Herpes simplex virus type 1 (HSV1) tegument proteins. A, HSV1 virions contain 25 tegument proteins, many of which are imported into the host nucleus (nuclear localization: green circle) and/or perform a nuclear function (bold) in HSV1 replication. pUS2 locates to the cytoplasm (cytoplasmic localization: rosy circle), while numerous proteins show a pancellular distribution (intersection green and rosy circle). Nuclear export analysis using the Nuclear EXport Trapped by RAPamycin (NEX‐TRAP) assay revealed that a group of nine HSV1 tegument proteins including pUL4,^12^ pUL7, pUL11, pUL13, pUL21, pUL37d11, pUL47, pUL48 and pUS2 exhibited nuclear export activity (yellow circle: NEX‐TRAP). B, To determine the native subcellular distribution of HSV1 tegument proteins in absence of other viral proteins, HeLa cells were transfected for 16 hours with a plasmid encoding a myc‐tagged tegument protein, incubated in absence (−LepB) or presence of Leptomycin B (+LepB) and subsequently analyzed by indirect immunofluorescence using mouse anti‐myc antibodies and secondary reagents. Nuclei were visualized by Dapi staining. The bar corresponds to 10 μm

Active transport of most proteins from the nucleus to the cytoplasm is mediated by the transport factor CRM1/Xpo1 and inhibited in presence of Leptomycin B. To determine the native subcellular distribution of all HSV1 tegument proteins and to independently validate their potential for CRM1/Xpo1 mediated nuclear export, myc‐tagged tegument proteins were transiently expressed in HeLa cells (Figure 2) in presence or absence of Leptomycin B. Their localization was classified into three categories N < C, N = C or N > C (Figure S1A) and validated in 100 transfected cells (Figure S1B). A large number of tegument proteins gained access to the nucleus (Figure 2A, green circle) and/or perform a function in this compartment (Figure 2A, bold). pUS2 exclusively located to the cytoplasm (Figure 2A, rosy circle), while the remaining seven proteins showed a pancellular distribution (Figure 2A, intersection green and rosy circle). Thus, with the exception of pUS2, all of the analyzed tegument proteins gained access to the nucleus potentially mediated by an intrinsic nuclear localization sequence (NLS).

All tegument proteins including the nine that showed nuclear export activity in the NEX‐TRAP assay were analyzed for their response to Leptomycin B (Figure 2B; Figure S1A). Myc‐tagged pUL4,^12^ pUL11, pUL13, pUL21 and pUL37d11 showed sensitivity to Leptomycin B consistent with the NEX‐TRAP results (Figure 2B; Figure S1B). pUL7, pUL47, pUL48 and pUS10 that had shown export activity using the NEX‐TRAP were exclusively localized in the nucleus precluding further nuclear retention (Figure 2B; Figure S1B). pUL49 and pUS2 were non‐responsive to Leptomycin B (Figure 2B; Figure S1B). In all other cases, the response to Leptomycin B was ambiguous potentially by indirectly influencing the host nuclear export (Figure 2B; Figure S1B). Together these data revealed that the five tegument proteins pUL4,^12^ pUL11, pUL13, pUL21 and pUL37d11 actively shuttle between nucleus and cytoplasm in a CRM1/Xpo1‐dependent fashion consistent with the presence of a functional leucine‐rich NES.

### 2.3 Bioinformatic prediction of nuclear export sequences in HSV1 tegument proteins

**Figure 3:**
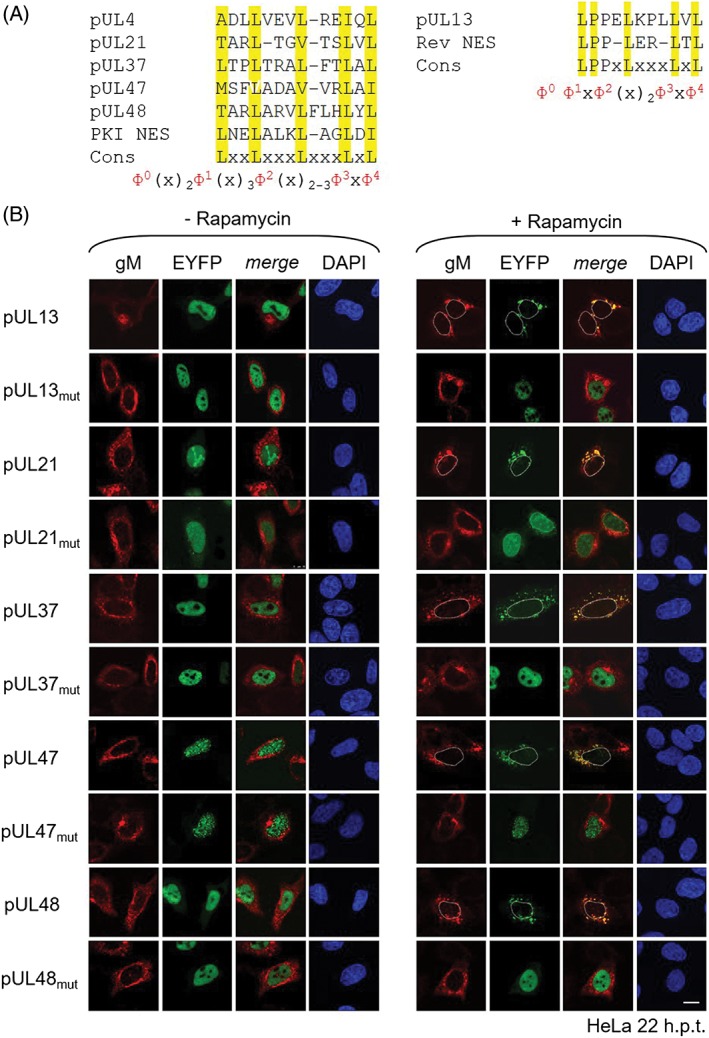
Identification of nuclear export sequences within the Herpes simplex virus type 1 (HSV1) tegument proteins. A, NES sequences identified by bioinformatic prediction^23^, ^26^, ^27^ were compared to the PKI NES or Rev NES and their consensus sequences. B, Identified NES sequences were mutagenized using site‐directed mutagenesis. To determine the nuclear export activity of wild‐type and NES mutant proteins, the Nuclear EXport Trapped by RAPamycin (NEX‐TRAP) was performed. HeLa cells were cotransfected for 20 hours with the plasmid pCR3‐N‐HA‐UL10/gM‐FKBP and a pEYFP‐NLS‐FRB plasmid encoding one of the analyzed HSV1 tegument proteins or mutants thereof. Following incubation of the cells with anisomycin and rapamycin, cells were analyzed by indirect immunofluorescence using rabbit anti‐gM antibodies and secondary reagents. EYFP‐tagged proteins were visualized directly. Nuclei were visualized by Dapi staining. The bar corresponds to 10 μm. Nuclei are marked by dashed lines

Bioinformatic prediction based on Reference 26 called NES pattern and (www.cbs.dtu.dk/services/NetNES 27) called NetNES had suggested a leucine‐rich nuclear export sequence (NES) within pUL4,^12^ pUL13, pUL21, pUL37d11, pUL47 and pUL48 while no NES was identified within pUL7, pUL11 and pUS2 (Table 2 bold; Table S2; Figure 3A). A third bioinformatic tool called LocNES (http://prodata.swmed.edu/LocNES/LocNES.php 23) was applied and revealed that—with few exceptions—most HSV1 tegument proteins are predicted to contain numerous leucine‐rich NESs varying in score (Table 2). Each of the NESs previously predicted within pUL4,^12^ pUL13, pUL21, pUL37d11, pUL47 and pUL48 using the NES pattern or NetNES algorithm was also identified by LocNES with a score >0.200, with the exception of the NES of pUL21 that had a rather low score of 0.077. Their comparison to the consensus sequences of the prototypic Rev and PKI NES (Figure 3A) showed that the NESs of pUL4,^12^ pUL21, pUL37d11, pUL47 and pUL48 all match the PKI NES and follow the consensus Ф^0^(x)_2_Ф^1^(x)_3_Ф^2^(x)_2‐3_Ф^3^xФ^4^. In contrast, the NES of pUL13 is characterized by two prolines at the N‐terminal end of LPPELKPLLVL reminiscent of a Rev NES and matches the consensus Ф^0^Ф^1^xФ^2^(x)_2_Ф^3^xФ^4^ with Ф^1^ = P >> L (Figure 3A). Furthermore, based on in silico analysis and structural data, most of the identified NESs are located in disordered and partial‐folded regions consistent with their accessibility to CRM1/Xpo1 and thus their functionality^17^ (Table S1). Together, the prediction of these NESs by at least two bioinformatic tools, their match with the NES consensus sequence of the PKI or Rev NES as well as their presence in disordered regions strongly suggested that they are functional.

**Table 2:** Nuclear export of HSV1 tegument proteins

HSV1 tegument	Other name/function	NES prediction using LocNES		*NES pattern* NetNES	Subcellular localization	LepB
		Sequence	Score			
RL1	ICP34.5	^134^LRLRVTAEHLARLRL^148^ (9)	0.749		N	+/−
RL2	ICP0	^738^NMLFDQGTLVGALDF^752^	0.272		N	+/−
**pUL4**		^172^ **PTADLLVEVLREIQL** ^186^ (11, 12)	0.547	^178^VEVLREIQL^186^	N	+
**pUL7**		^132^NPRDLLDFELACLLM^146^	0.306		N	Nd
**pUL11**		^22^GEVVSLTAHDFDVV^36^	0.220		NC	+
**pUL13**		^492^ **LPPELKPLLVLVSRL** ^506^	0.440	^*492*^ *LPPELKPLLVL* ^*502*^	NC	+
pUL14		^136^AEGWMSPADSDLLVM^150^	0.380		N	+/−
pUL16		^68^RCLTVLTEPLCQVAL^82^ ^132^SVTHPLTPLLCTLTF^146^	0.333 0.352		N	+/−
**pUL21**		^476^ **ATHTARLTGVTSLVL** ^490^ (this study); exp. NES has low score	0.077	^*482*^ *LTGVTSLVL* ^*490*^	N	+
pUL23	TK	^38^TEVRLEQKMPTLLRV^52^	0.307		N	+/−
pUL36a/b (a: aa 1‐1000; b: aa 1001‐2000)	Large tegument	^36^GSVSCMRSSLSFLSL50 ^376^DDDDMRVLEMGVVPV^390^ ^630^ENSLIGRLALAKLIL^644^ ^826^AAVVPVVQLLESLPV^840^ ^2311^ARVTAMDLVLAAVLL^2325^	0.369 0.317 0.328 0.525 0.240	^*639*^ *LADVAAHLPL* ^*648*^	NC	+/−
**pUL37d11** (aa 12‐1124)	ICP32	^22^SDGPMQRLLASLAGL^36^ ^226^FPAPFVQEGLRFLAL^240^ ^258^ **ATLTPLTRALFTLAL** ^272^ (7; this study) ^695^MADNIEQLLRELYVI^709^ ^755^RADPLIRQLEDAIVL^769^ ^763^LEDAIVLLRLHMRTL^777^	0.281 0.346 0.488 0.413 0.243 0.620	^*263*^ *LTRALFTLAL* ^*272*^	NC	+
pUL41	Vhs	^30^PIAVDLWNVMYTLVV^44^ (10); very low score	0.038		N	+/−
pUL46a/b (a: aa 1‐336; b: aa 337‐719)	VP11/12	^320^GTVDTVVSTVEVLSI^334^	0.239		N	+/−
**pUL47**	VP13/14	^282^ **QAMSFLADAVVRLAI** ^296^ (this study) ^314^LDDRAAELRRQFASL^328^ ^651^ **PRVRVVDIMSQFRKL** ^665^ (8)	0.212 0.228 0.247	^*287*^ *LADAVVRLAI* ^*296*^	N	Nd
**pUL48**	VP16/ICP25	^57^PPAALFNRLLDDLGF^71^ ^220^ **ARLARVLFLHLYLFL** ^234^ (this study)	0.244 0.364	^*222*^ *LARVLFLHLYL* ^*232*^	N	Nd
pUL49	VP22	^229^DEDLNELLGITTIRV^243^	0.342		N	−
pUL50	dUTPase	^117^FAPGTLRVDVTFLD^131^	0.286	^*122*^ *LRVDVTFLDI* ^*131*^	N	+/−
pUL51		^142^RSMAESDVVMEDVAI^156^	0.314		NC	+/−
pUL55		^1^MTATPLTNLFL^11^	0.413	^96^LRELEDKRGVRL^107^	N	+/−
RS1	ICP4	^625^AWLRELRFVRDALVL^639^ ^1003^TRDLAFAGAVEFLGL^1017^	0.823 0.292	^*630*^ *LRFVRDALVL* ^*639*^	N	+/−
**pUS2**		No prediction			C	−
pUS3		^236^RLDHPAILPLLDLHV^250^ ^461^FDGALRPSAAELLCL^475^	0.352 0.500		NC	+/−
pUS10		^170^GLYPLDARALAHLVM^184^	0.412	^174^LDARALAHLVM^185^	N	nd
pUS11		No NES candidate predicted			NC	+/−

Abbreviations: +/− responsivity to Leptomycin B; C, cytoplasmic; N, nuclear; NC, nucleo‐cytoplasmic localization; nd, not determined.

Only predictions with scores >0.200 (except for pUL21 and pUL41) are shown. Tegument proteins with export activity based on NEX‐TRAP are shown in bold; NESs identified by NEX‐TRAP are bold with consensus amino acids underlined. The three proteins RL1, RL2, and RS1 could not be analyzed since their coding sequence was unavailable.

### 2.4 Verification of NESs within HSV1 tegument proteins

To verify the NES sequences identified in pUL4,^12^ pUL13, pUL21, pUL37d11, pUL47 and pUL48 using bioinformatic predictions, they were individually mutated by replacing the three last consensus residues (Ф) to alanines (eg, Lx_2‐3_ Lx_2_ LxL to Lx_2‐3_Ax_2_AxA; Table 2). To compare the nuclear export activity of the NES mutant and wild‐type proteins, the NEX‐TRAP assay was again applied (Figure 3B). Upon addition of Rapamycin, all wild‐type proteins were recruited to the TGN confirming the previous results (Figure 3B, right panel; Figure 1, right panel). In contrast, all proteins with NES mutations remained in the nucleus and thus were unable to reach the TGN for FRB‐FKBP‐complex formation. Thus, each of the tegument proteins pUL4,^12^ pUL13, pUL21, pUL37d11, pUL47 and pUL48 contains a single leucine‐rich NES required for nuclear export. Since pUL11, like pUL13 and pUL37d11, responds to Leptomycin B (Figure 2B), it likely interacts with CRM1/Xpo1 based on a so‐far unknown leucine‐rich NES while the mode of nuclear export of pUL7 and pUS2 remains to be identified.

### 2.5 Nuclear export activity of EBV tegument proteins

**Figure 4:**
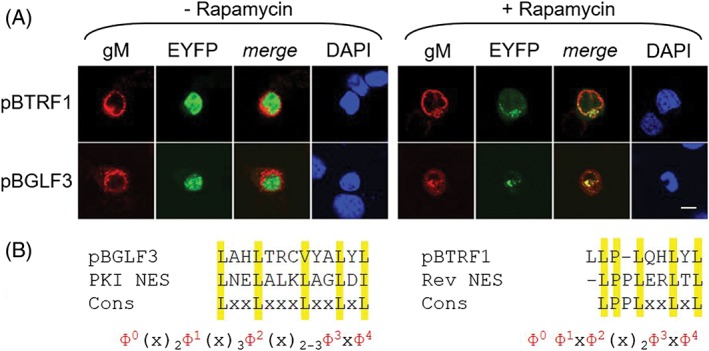
Conservation of nuclear export between HSV1 and EBV. A, Nuclear export of EBV tegument proteins as determined by Nuclear EXport Trapped by RAPamycin (NEX‐TRAP). HeLa cells were co‐transfected with the plasmid pCR3‐N‐HA‐UL10/gM‐FKBP and the pEYFP‐NLS‐FRB plasmid encoding individual EBV proteins and the NEX‐TRAP assay was performed 20 hours after transfection. The transfected cells were treated with anisomycin/rapamycin, fixed and immunostained with anti‐gM and fluorophore‐labeled secondary antibody. EBV proteins were detected by direct visualization of the EYFP‐tag. Nuclei were counterstained with Dapi. The bar corresponds to 10 μm. B, EBV NES sequences were identified by bioinformatic prediction and compared to the PKI or Rev NES and their consensus sequences (Cons)

In order to test whether orthologous tegument proteins share the ability to shuttle between nucleus and cytoplasm, EBV orthologs of actively exported HSV1 tegument proteins were included in the study. EBV was chosen as a representative of a different herpesvirus subfamily that has a similar coding capacity as HSV1. Out of the nine exported HSV1 proteins, five are evolutionarily conserved (Table 1). Known orthologs in EBV^28^, ^29^ (Table 1) include pUL7 (pBBRF2), pUL11 (pBBLF1), pUL13 (pBGLF4), pUL21 (pBTRF1) and pUL37 (pBOLF1). All of these EBV proteins (except for pBOLF1 that was not tested in this study) were previously reported to show nuclear as well as cytoplasmic localization^5^ which is in agreement with nucleo‐cytoplasmic trafficking. In the NEX‐TRAP assay, pBBRF2 (pUL7) localized to both the nucleus and the cytoplasm prior to addition of Rapamycin, and pBBLF1 (pUL11) was localized to defined spots within the cytoplasm (Figure S2). Since these proteins could not be forced into the nucleus by the SV40‐NLS, their nucleo‐cytoplasmic shuttling cannot be determined by NEX‐TRAP analysis (Figure S2). The remaining EBV proteins pBGLF4 (pUL13) and pBTRF1 (pUL21) behaved differently. While pBGLF4 remained nuclear in presence of Rapamycin, pBTRF1 co‐localized with the gM‐FKBP at the TGN in presence of Rapamycin (Figure 4A and data not shown). Thus, unlike its HSV1 ortholog pUL13, pBGLF4 did not show any export activity, while pBTRF1 appears to be actively exported from the nucleus as is its HSV1 ortholog pUL21.

Next, nuclear export was analyzed for EBV orthologs pBGLF3, pBGLF2 and pBLLF3 of three non‐exported HSV1 tegument proteins: pUL14, pUL16 and pUL50, respectively (Figure 1, Table 1). These HSV1 and EBV proteins have been reported to show either pan‐cellular or nuclear localization in transfection experiments, indicating that all three proteins access the nucleus.^5^ Upon NEX‐TRAP analysis, all three EBV proteins accumulated in the nucleus in absence of Rapamycin (Figure S2; Figure 4A). While pBGLF2 and pBLLF3 remained in the nucleus in presence of Rapamycin as were their HSV1 orthologs (Figure S2; Figure 1), pBGLF3 was translocated to the TGN (Figure 4A). Thus, pBGLF3 is exported from the nucleus in contrast to its non‐exported HSV1 ortholog pUL14.

To identify putative NESs in the EBV tegument proteins, three bioinformatic tools used for prediction of NESs within HSV1 tegument proteins were applied (Table 3).^23^, ^26^, ^27^ As expected based on the HSV1 analysis, the LocNES identified numerous potential NESs in essentially each EBV tegument protein (Table 3). In contrast, the NES pattern (but not the NetNES), revealed NESs within 10 tegument proteins. For both pBGLF3 and pBTRF1, that had shown export activity upon NEX‐TRAP analysis, a candidate NES could be identified using two bioinformatics tools, that in either case scored lowest of all NESs predicted by LocNES (Table 3). The putative NES of pBGLF3 matches the PKI NES (consensus Ф^0^(x)_2_Ф^1^(x)_3_Ф^2^(x)_2‐3_Ф^3^xФ^4^) while the putative pBTRF1 NES matches the consensus of the Rev NES (Ф^0^Ф^1^xФ^2^(x)_2_Ф^3^xФ^4^; Figure 4B; Table 3). In conclusion, putative NESs that resemble known functional NESs could be identified in both exported EBV proteins combining experimental data with at least two bioinformatic prediction algorithms. Taken together, nuclear export activity of tegument proteins is only partially conserved between HSV1 and EBV.

**Table 3:** Nuclear export of EBV tegument proteins

EBV tegument	Function^29^	NES prediction LocNES		NES pattern
		Sequence	Score	
pBALF2	ssDNABP	^529^QLNQNLLERLSRLGI^543^ ^611^CPIFTQLFYRSLLTI^625^ ^617^LFYRSLLTILQDISL^631^ ^621^SLLTILQDISLPICM^635^	0.611 0.444 0.292 0.233	^*534*^ *LLERLSRLGI* ^*543*^
*pBBLF1*	MYrPBP	No NES candidate >0.200 predicted		
*pBBRF2*		^16^TMQKVSLRVTPRLVL^30^ ^127^PSIGLCREVLGRLTL^141^	0.229 0.292	^*131*^ *LCREVLGRLTL* ^*141*^
pBDLF2		^126^GGQRGAPISADLLSL^140^ ^192^IYYCCYLAFLALLAF^206^ ^194^YCCYLAFLALLAFGF^208^ ^197^YLAFLALLAFGFNPL^211^ ^199^AFLALLAFGFNPLFL^213^	0.420 0.254 0.161 0.172 0.195	^*204*^ *LAFGFNPLFL* ^*213*^
pBFRF1		^314^TPYLARVLAVTAVAL^328^ ^316^YLARVLAVTAVALLL^330^ ^317^LARVLAVTAVALLLM^331^ ^318^ARVLAVTAVALLLMF^332^ ^319^RVLAVTAVALLLMFL^333^	0.268 0.461 0.394 0.319 0.225	
pBGLF1	Packaging	^105^EGGLVGELQIYYLSL ^170^QVEHPKTYDLKQILL^184^ ^172^EHPKTYDLKQILLEI^186^	0.299 0.467 0.373	
*pBGLF2*	*MYrPBP*	^187^PNMPTFPSLTHFINL^201^ ^188^NMPTFPSLTHFINLL^202^	*0.164* *0.172*	
**pBGLF3**		^33^TPEQFKLVETPLKSF^47^ ^35^EQFKLVETPLKSFLL^49^ ^36^QFKLVETPLKSFLLV^50^ ^73^DDYDFSSLALELLPL^87^ ^**207**^ **AFLAHLTRCVYALYL** ^**221**^	0.217 0.265 0.226 0.630 **0.163**	^*212*^ *LTRCVYALYL* ^*221*^
*pBGLF4*	ST kinase	^50^LKVTNIDDMTETLYV^64^ ^102^KLYDSVTELYHELMV^116^ ^348^EVLSQMWNLNLDMGL^362^	0.248 0.207 0.150	^*376*^ *LSQMWNLNL* ^*384*^
pBKRF4		No NES candidate predicted		
*pBLLF3*	*dUTPase* ^*5*^	^14^DKLLLQQASVGRLTL^28^ ^130^GDVGLDVSLPKDLAL^144^	*0.263* *0.100*	^*134*^ *LDVSLPKDLAL* ^*244*^
pBLRF2		^15^VDMSMEDMAARLARL^29^ ^99^TTRNEMENILQNLTL^113^ ^103^EMENILQNLTLRIQV^117^	0.263 0.600 0.479	
pBMRF1	dsDNABP	^240^VCSVAADSLAAALSL^254^ ^274^AVVAGLLTSAGDLPL^288^ ^278^GLLTSAGDLPLDLSV^292^ ^279^LLTSAGDLPLDLSVI^293^ ^281^TSAGDLPLDLSVILF^295^	0.233 0.613 0.483 0.329 0.201	
pBNRF1	MTP	^49^LGLDPGPLIAENLLL^63^ ^52^DPGPLIAENLLLVAL^66^	0.684 0.303	
pBOLF1	LTPBP	^24^EVDGGLARVTRQLLL^38^ ^39^SGDDPAARLRALMPL^53^ ^41^DDPAARLRALMPLEL^55^ ^43^PAARLRALMPLELGI^57^ ^46^RLRALMPLELGIFGL^60^ ^64^AQPVLVRDFLNTLTL^78^ ^304^AAVPVLAFDAARLRL^318^ ^414^RWRWLEATAALLESL^428^ ^424^LLESLSGFALHFFRL^438^	0.053 0.216 0.257 0.219 0.221 0.333 0.334 0.207 0.242	^*29*^ *LARVTRQLLL* ^*38*^
pBORF2	RNR‐L	^428^TQGDELLLALPRLSV^442^ ^734^DLGVMECKASAALSV^748^	0.303 0.217	
pBPLF1	LTP	^732^PEMDFVPLESNIARI^746^ ^814^PTLDLEQLLTSELNI^828^ ^1117^NEKRLRTILDDIEAM^1131^ ^1120^RLRTILDDIEAMLGL^1134^ ^1289^ATHSTLKETAAAVNL^1303^ ^1485^NTDILDSLTQILAAM^1499^ ^1487^DILDSLTQILAAMLL^1501^ ^1489^LDSLTQILAAMLLGI^1503^ ^1848^GMAQNSMDGMEELRL^1862^ ^1853^SMDGMEELRLALATL^1867^ ^3063^VVTQFLSIEDIIREV^3077^ ^3130^LERSTHRLIADLERL^3144^ ^3132^RSTHRLIADLERLKF^346^ ^3135^HRLIADLERLKFLYL^3149^	0.251 0.398 0.350 0.569 0.252 0.234 0.327 0.420 0.399 0.408 0.203 0.530 0.738 0.636	^*818*^ *LEQLLTSELNI* ^*828*^
pBRRF2		^207^VFARTLLAALFHLNM^221^ ^211^TLLAALFHLNMFFIL^225^ ^473^QDANLEDVQREFSGL^487^ ^475^ANLEDVQREFSGLRV^489^	0.323 0.401 0.613 0.631	
pBSRF1	PalmP	No NES candidate >0.200 predicted		
**pBTRF1**		^59^ESLPMTNMRAPIISL^73^ ^64^TNMRAPIISLARLIL^78^ ^169^DILEIPVTVLSSLQL^183^ ^247^ **LVPNPDLLPLQHLYL** ^261 252^ **DLLPLQHLYLKHVVL** ^266^	0.259 0.448 0.229 0.171 0.314	^*274*^ *LLPLQHLYL* ^*282*^
pBXLF1	TK	^165^MYQKGFEEGLAGLGL^179^ ^235^QLKRLSGSVMNVLNL^249^ ^392^DRHLLSASVVFPLML^406^ ^541^EVCLAFTRTLAYLQF^555^	0.477 0.356 0.209 0.285	^*239*^ *LSGSVMNVLNL* ^*249*^

Only LocNES predictions with scores >0.200 (except for pBBLF1, pBDLF2, pBGLF2, pBGLF3, pBGLF4, pBLLF3, pBOLF1 and pBTRF1) are shown. No predictions with NetNES. Tegument proteins with export activity based on NEX‐TRAP are bold. For those, NESs identified by at least two bioinformatic tools are bold with consensus amino acids underlined. Italics: proteins not exported in the NEX‐TRAP assay; pBBLF1 and pBBRF2 were subjected to NEX‐TRAP analysis but nuclear export could not be evaluated.

### 2.6 Comparative analysis of nucleo‐cytoplasmic trafficking of HSV1 and EBV proteins

**Figure 5:**
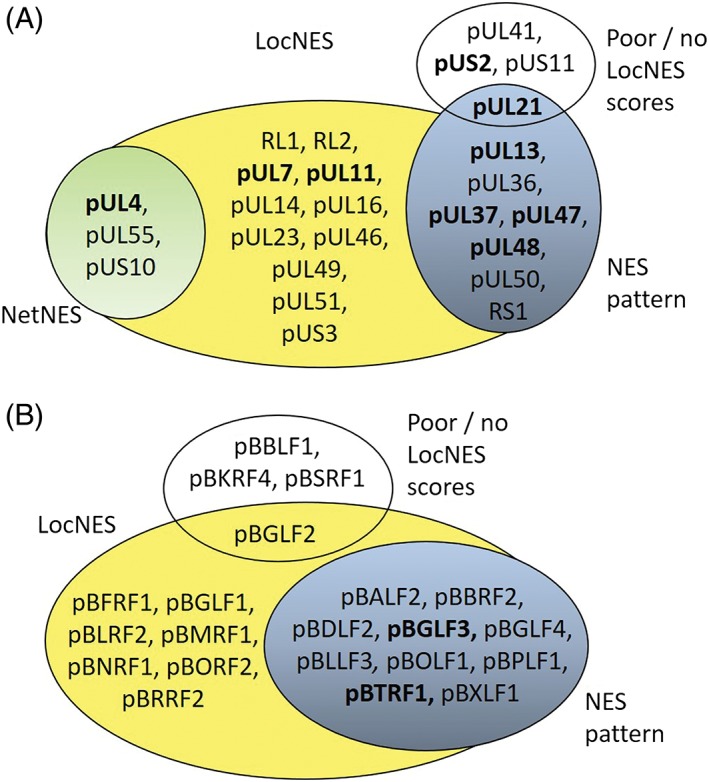
Comparison of bioinformatic NES predictions and Nuclear EXport Trapped by RAPamycin (NEX‐TRAP) activity of HSV1 and EBV tegument proteins. HSV1 (A) and EBV (B) tegument proteins as predicted by NetNES,^27^ by NES pattern,^26^ LocNES^23^ and determined by NEX‐TRAP.^12^ High (>0.200) and no/poor (<0.200) LocNES scores are indicated. Bold: Proteins exported in the NEX‐TRAP assay

Bioinformatic prediction of NESs is thought to be difficult due to a high degree of variability regarding the identity and spacing of hydrophobic amino acids in experimentally confirmed functional NESs. Initially, NES prediction was limited to two bioinformatic tools, the NES pattern^26^ and the NetNES (www.cbs.dtu.dk/services/NetNES 27). More recently, other bioinformatic tools were developed^19^, ^20^, ^21^, ^22^ including the LocNES algorithm (http://prodata.swmed.edu/LocNES/LocNES.php 23). Comprehensive analysis of all HSV1 tegument proteins using the LocNES revealed that, with few exceptions (pUL41, pUS2, pUS11), most HSV1 tegument proteins were predicted to contain a leucine‐rich NES with a score >0.200 (Table 2; Figure 5A). The NES pattern analysis^26^ identified a leucine‐rich NES within eight HSV1 proteins. In contrast, only three proteins (pUL4, pUL55, pUS10) were predicted to contain a NES using the NetNES algorithm (Table 2; Figure 5A). No overlap exists between the NES pattern and the NetNES. HSV1 proteins shown to actively shuttle between nucleus and cytoplasm using the NEX‐TRAP were present in each of the three groups of predictions but more frequent in the NES pattern prediction^26^ (Table 2; Figure 5A, bold).

Similarly, all EBV tegument proteins were systematically analyzed using nuclear export prediction tools (Table 3; Figure 5B). The NES pattern identified a NES in 10 of the investigated proteins, while no prediction was achieved using NetNES. Using the LocNES, NESs were predicted in most EBV tegument proteins sometimes with rather low scores (Table 3), like for pBBLF1 (pUL11), pBSRF1, pBKRF4 and pBGLF2. In the remaining 17 EBV proteins, LocNES predicted at least one NES with a score >0.200 (Table 3). For both EBV tegument proteins exported in the NEX‐TRAP assay, a NES was identified by two prediction tools, the NES pattern and the LocNES. The NES predicted for pBGLF3 had a low LocNES score of 0.163 while that of pBTRF1 achieved a high score of 0.314 (Table 3, bold; Figure 5B, bold). pBGLF2 that was not exported in the NEX‐TRAP assay and pBBLF1 that was excluded from the nucleus reached only poor scores or no NES prediction, respectively (Figure S2). Together, this suggests that the score level may indicate the level of confidence in the prediction, however, a second prediction tool like the NES pattern^26^ (Figure 5B) strongly facilitates the identification of candidate NESs for further validation. Thus, bioinformatic prediction of nuclear export activity appears to be insufficient and there is still an essential need for experimental validation of predicted NESs by a suitable assay such as the NEX‐TRAP assay.

### 2.7 Functional characterization of the nuclear export activity of pUL48/VP16

**Figure 6:**
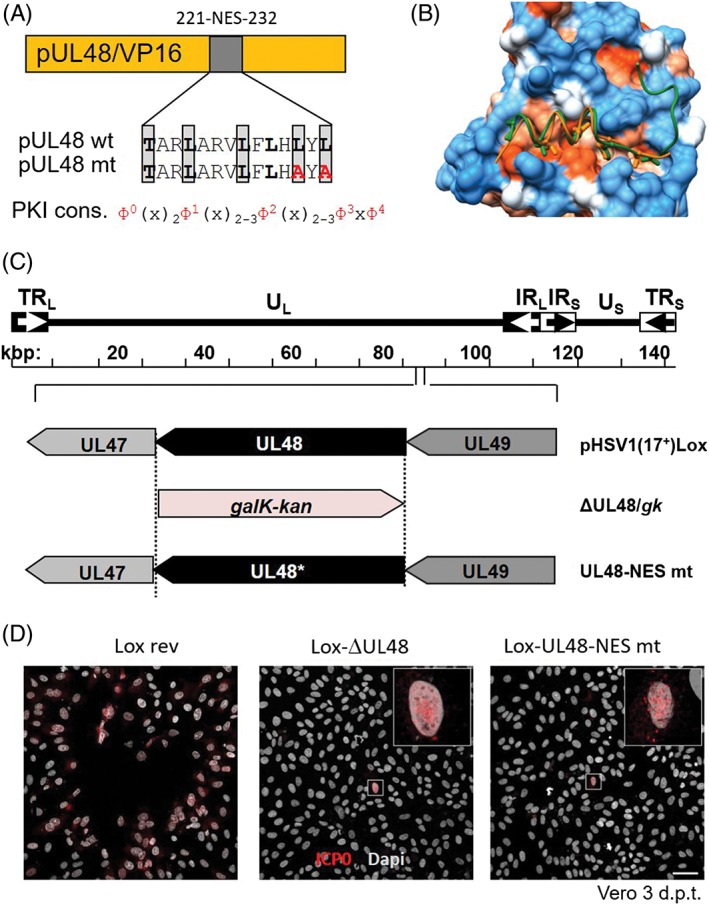
The pUL48/VP16 NES is important for Herpes simplex virus type 1 (HSV1) replication. A, Grafical depiction of pUL48/VP16 with the wild‐type and mutant 221‐NES‐232 compared to the PKI NES consensus sequence. B, Structural representation of the pUL48/VP16‐NES in complex with CRM1 based on the structural model of the Snurportin 1‐NES. C, A schematic diagram of the pHSV1(17+)Lox genome as well as the strategy to replace the UL48 coding region with the *galK‐kan* selection cassette resulting in Lox‐ΔUL48/*galk‐kan* is depicted. The *galK‐kan* cassette was replaced by the wt or NES mt UL48 sequence. D, The ability of the pHSV1(17+)Lox‐UL48 wild‐type or mutant to form plaques was tested by transfecting Vero cells with BAC DNA and scoring cytopathic effects at 3 days post transfection. To visualize transfected cells, indirect immunofluorescence analysis using anti‐ICP0 antibodies followed by Alexa 555‐conjugated secondary antibodies was performed. Insets contain magnifications of individual ICP0‐positive cells. The scale bar corresponds to 50 μm

The nuclear export activity of the HSV1 tegument protein pUL48/VP16, an essential protein involved in immediate early transcriptional activation (Reference 3; and references therein), was analyzed in more detail. The identified NES of pUL48/VP16 is located between residues 221 to 232 (Figure 6A). To reveal the conformation of the pUL48/VP16 NES upon binding to the CRM1/Xpo1 groove, a structural representation was performed based on the model of the Snurportin 1‐NES in complex with CRM1 (PDB ID 3NBY^30^). As expected for a NES with a PKI‐like consensus sequence, the pUL48/VP16 NES is mostly α‐helical (Figure 6B).

To analyze the function of the pUL48/VP16 NES in the context of an HSV1 infection, we generated the BAC mutant pHSV1(17+)Lox‐UL48‐NES where the pUL48/VP16 wild‐type NES TARLARVLFLHLYL was replaced by the NES mutant sequence TARLARVLFAH**A**Y**A** (Figure 6A, C; Table 2). A rescue mutant was generated for pHSV1(17+)Lox‐ΔUL48/*galK‐kan* resulting in Lox‐UL48‐NES *rev*. All mutations were verified by restriction digest and sequencing of the mutated regions of the BAC DNAs. Next, the BAC DNAs of the respective mutants or the parental pHSV1(17+)Lox were transfected into Vero cells (Figure 6D). pHSV1(17+)Lox rev readily formed plaques surrounded by cells expressing the HSV1 immediate early protein ICP0. Consistent with an essential function of HSV1 pUL48/VP16^31^, transfection of pHSV1(17+)Lox‐ΔUL48/*galK‐kan* resulted in single cells expressing ICP0 while no plaques were formed. Transfection of the pHSV1(17+)Lox‐UL48‐NES mt also resulted in single cells positive for ICP0 but no plaques (Figure 6D), suggesting that the leucine‐rich NES of pUL48/VP16 and thus nuclear export represents an important feature of this protein.

## 3 DISCUSSION

Using the previously established NEX‐TRAP assay, we performed a comprehensive analysis of nuclear export activity within HSV1 tegument proteins packed into HSV1 virions.^1^ Only few HSV1 proteins including RL1, RL2, RS1, as well as the pUL36 middle and C‐terminal region remained uncovered. Out of 22 tegument proteins analyzed (Reference 12; this data), 9 exhibited nuclear export activity, 5 of which (pUL7, pUL11, pUL13, pUL21, pUL37) are evolutionarily conserved (Table 1). Nuclear export activity was confirmed within HSV1 pUL4^11^, ^12^ and HSV1 VP13/14/pUL47 (Reference 8 and references therein), and newly identified within HSV1 pUL37d11 confirming data in HSV2.^7^ Tegument proteins previously not known to actively exit the nucleus, but implied to function in both nucleus and cytoplasm, include pUL7, pUL11, pUL21, VP16/pUL48 and pUS2. The kinase pUL13 with substrates in both nucleus and cytoplasm was also found to shuttle. Deletions of most of them lead to severe growth defects consistent with their important functions during HSV1 replication.

This and our previous analysis^12^ identified a leucine‐rich NES within six tegument proteins including pUL4, pUL13, pUL21, pUL37d11, pUL47 and pUL48. Mutational analysis showed that each of these tegument proteins carries a single NES sufficient to mediate its nuclear export. Independent evidence for nuclear export activity of pUL4, pUL11, pUL13, pUL21 and pUL37d11 was provided by Leptomycin B treatment that changed their subcellular distribution to a predominantly nuclear one, indicating they all contain a leucine‐rich NES. Furthermore, in the NEX‐TRAP, HSV1 protein pUL7 and pUS2 showed nuclear export activity. Yet, neither of these proteins contains an obvious leucine‐rich NES. Potentially these proteins shuttle in a CRM1/Xpo1 independent fashion, however, further analysis is necessary to validate these findings. Most actively exported tegument proteins including pUL4, pUL21, pUL37d11, pUL47 and pUL48 harbor a NES that matches the consensus Ф^0^(x)_2_Ф^1^(x)_3_Ф^2^(x)_2‐3_Ф^3^xФ^4^ of the PKI NES. The NES LPPELKPLLVL of pUL13 is exceptional in that it contains two prolines reminiscent of the Rev NES and matching the consensus sequence Ф^0^Ф^1^xФ^2^(x)_2_Ф^3^xФ^4^. Thus, HSV1 proteins contain two different types of NESs divergent in sequence and classified as Rev‐ and PKI‐NES‐like elements.

Based on our results, we suggest a sequence of approaches to generally identify and characterize the nuclear export of proteins: (a) determine nuclear export activity by using the NEX‐TRAP, and/or another experimental approach, (b) identify a potential NES by an overlap of at least two different in silico prediction tools and (c) mutationally analyze the predicted NES in the context of the full‐length protein and experimentally verify the loss of nuclear export. As shown here, this approach allowed us to determine nuclear export activity within pUL4, pUL13, pUL21, pUL37d11, pUL47 and pUL48. Furthermore, targeted mutagenesis of each of the bioinformatically predicted NESs abolished nuclear export of the respective full‐length protein. One should, however, keep in mind that mutating hydrophobic residues of a putative NES may disrupt the overall conformation of a given protein thereby disrupting various functions including the binding to a partner protein that may be critical for indirect nuclear export. To avoid the misidentification of NESs as previously reported,^24^, ^32^, ^33^ further validation is important. To this end, the ability of a peptide NES to mediate nuclear export of an unrelated protein, and to bind CRM1/Xpo1 in a Ran‐GTP‐dependent manner can be tested.

The NESs identified within pUL4^11^, ^12^ and pUL37d11^7^ match the previously identified signal sequence. An interesting discrepancy was observed for pUL47 (Reference 8; and references therein). Both datasets agree that pUL47 shuttles between nucleus and cytoplasm in a CRM1/Xpo1‐dependent manner using a leucine‐rich NES. However, two different peptides both matching the PKI consensus NES were found responsible for active nuclear export. The NES between residues 647 and 670 was identified to mediate nuclear export of a fragment of pUL47 while at the same time, the NES between residues 282 and 296 identified by our study remained undiscovered.^8^ One explanation of this discrepancy is the fragmentation of pUL47 with each fragment hooked up to GFP‐SV40‐NLS.^8^ The strong SV40‐NLS may have led to a predominantly nuclear localization of the NES (282‐296) fusion protein masking its potential nuclear export activity. The NES identified by our study, however, is responsible for pUL47 nuclear export in the protein context and its targeted mutagenesis abrogated nuclear export of the full‐length pUL47. Most interestingly, the previous study showed that nuclear export of pUL47 occurs in the viral context.^8^ Thus, with the single NES identified by our study, the functional significance of pUL47 nuclear export can now be determined by targeted mutagenesis of the NES in the viral context.

Analysis of this large group of viral tegument proteins demonstrates the value and applicability of the recently established NEX‐TRAP assay.^12^ This assay clearly simplifies and allows for the in vivo detection of nuclear export of proteins in their native conformation. A prerequisite of this assay is the constitutive nuclear import of proteins to be analyzed making nuclear export activity of proteins independent of their intrinsic nuclear import activity. Thus, proteins located in both the cytoplasm and nucleus, for example, due to cytoplasmic retention are poorly suited for NEX‐TRAP analysis. This is the case for HSV1 vhs/pUL41 previously reported to contain export activity.^10^ In case of the HSV1 tegument protein pUL50, a distinct cytoplasmic pool was observed that was recruited to the gM‐FKBP reporter upon addition of Rapamycin. At the same time, the pool of nuclear pUL50 remained unaltered suggesting that the NES predicted by Reference 26 is nonfunctional. Another limitation is imposed by viral proteins that may be natural partners of gM. Like Pseudorabies virus pUL49,^34^ HSV1 pUL49 may interact with gM and thus associate with the gM‐FKBP reporter prior to addition of Rapamycin. Instead of gM‐FKBP, a non‐viral FKBP‐fused transmembrane reporter protein could be used to determine a putative export activity. Interestingly, pUS3 was negative for nuclear export based on the NEX‐TRAP assay but sensitive to Leptomycin B. This discrepancy may be explained by localization of pUS3 to dense nuclear bodies thereby retaining the protein in the nucleus. Therefore, pUS3 that contains two potential NESs with high scores (Table 5) is a potential shuttling protein that escaped the NEX‐TRAP analysis due to nuclear retention.

In this work, several NES prediction algorithms including NetNES,^27^ LocNES^23^ and NES pattern^26^ were applied to systematically scan HSV1 and EBV tegument proteins. The three algorithms concordantly search for leucine‐rich sequence elements indicative of CRM1/Xpo1‐dependent nuclear export. However, they differ substantially, for example, in the underlying NES consensus sequences and in the consideration of additional information such as flanking or background sequences. Consequently, the results deviated considerably between the three algorithms. The two more stringent algorithms NetNES and NES pattern predicted several NESs experimentally confirmed in the NEX‐TRAP assay but in some cases also failed, for example, HSV1 pUL7 and pUL11. On the other hand, the most recent of the three algorithms, LocNES,^23^ was also the least stringent and predicted NESs in almost all proteins that were scanned which raises the question if and how many false‐positive NESs might be included in the LocNES results. At least for HSV1 tegument proteins, LocNES predicted several NESs that clearly were nonfunctional in the NEX‐TRAP assay such as pUL14, pUL16 or pUL51. Even the most stringent algorithm, NetNES, predicted NESs in two nonexported HSV1 proteins, pUL55 and pUS10, while it correctly identified only one exported protein, pUL4. Strikingly, none of the confirmed exported HSV1 proteins was identified by all three algorithms. The consequence of these observations is that bioinformatic NES prediction to date is still insufficient to reliably identify or exclude functional NESs likely influenced by the fact that NESs are structurally divergent and flexibly fit the CRM1/Xpo1 binding pocket.^17^ Hence, experimental identification and validation is mandatory. However, since mutational analysis of HSV1 NESs predicted by the NES pattern and by NetNES confirmed their functionality in all cases, the major strength of a range of developed NES prediction algorithms^19^, ^20^, ^21^, ^22^, ^23^ may currently lie in the detection of the relevant export signals in proteins known to be exported.

In order to examine to what extent the nuclear export activity of herpesviral tegument proteins is conserved, EBV orthologs of HSV1 proteins analyzed with the NEX‐TRAP assay were tested. pBGLF2 and pBLLF3 fusion proteins and their HSV1 orthologs maintained nuclear localization in presence of Rapamycin. Both pBLLF3 and its HSV1 ortholog pUL50 were predicted to have a NES based on NES pattern.^26^ Both proteins were reported previously to be functional dUTPases,^35^, ^36^, ^37^ additionally the EBV dUTPase was shown to be localized predominantly to the nucleus.^38^, ^39^ Thus, the NEX‐TRAP result suggesting that pUL50 and pBLLF3 are not actively exported from the nucleus is plausible and in agreement with a nuclear function and with the pre‐existing data. We found two EBV tegument proteins to be exported from the nucleus in the NEX‐TRAP assay. The nuclear export of pBGLF3 was in contrast to its non‐exported HSV1 ortholog pUL14. In the study by Reference 5, pBGLF3 was localized both in the nucleus and in the cytoplasm which is in line with our data. To our knowledge, the functional data available for pBGLF3 is limited to a report suggesting a role in late viral gene regulation.^40^ It remains to be seen how nucleo‐cytoplasmic shuttling contributes to this protein's function. The second exported EBV protein, pBTRF1 and its HSV1 ortholog pUL21 were localized in both nucleus and cytoplasm,^5^ and both were found to be actively exported in the NEX‐TRAP assay. Although the functional NES in HSV1 pUL21 rather resembles the PKI NES and lacks prolines, it shares the overall structure with its EBV ortholog pBTRF1, that can be expressed by the Rev NES consensus Ф^0^Ф^1^xФ^2^(x)_2_Ф^3^xФ^4^. Altogether, the capacity for nucleo‐cytoplasmic shuttling appears to be only partially conserved in orthologous tegument proteins of HSV1 and EBV. This emphasizes the need for experimental determination of the export activity of individual proteins, for example, by NEX‐TRAP analysis.

One of the HSV1 tegument proteins was analyzed in more detail, VP16/pUL48, an essential protein that is part of the infectious particle and involved in immediate early transcriptional activation prior to viral gene expression (Reference 3; and references therein). The VP16 NES was identified and validated by the NEX‐TRAP assay. Subsequent site directed mutagenesis of the VP16 NES in the context of the BAC HSV1(17^+^)Lox resulted in a defect in viral replication. Together these data revealed a yet unknown, important function embedded within pUL48/VP16 for viral propagation, further detailed analysis is required to determine at which step of the viral life cycle nuclear export of pUL48/VP16 is required.

Our analysis identified nuclear export activity within several HSV1 and EBV tegument proteins. Thus, the question arises why nuclear export could be important for these tegument proteins. Several tegument proteins of the virion perform a nuclear function immediately after entry into the cell, such as transcriptional activation of viral genes, for example, VP16/pUL48. Others are known or suggested to modulate nuclear functions throughout the infection cycle, for example, pUL13 and pUS3. This requires that they reach the nucleus either by an intrinsic NLS or by piggy‐backing on other viral or host proteins. Export of a viral tegument protein from the infected nucleus may subsequently become important (a) to regulate or finalize its nuclear activity, (b) to transport associated molecules out to the cytoplasm, (c) to perform an additional function in the cytoplasm or (d) to co‐ordinate nuclear and cytoplasmic events. The functional relevance of nuclear shuttling of tegument proteins has so far only been reported for VP13/14/pUL47.^8^, ^10^ With this study on the nuclear shuttling activity and the NESs identified within a range of tegument proteins it is now possible to decipher the molecular details of nuclear export.

## 4 MATERIALS AND METHODS

### 4.1 Cells, plasmids and viruses

HeLa (ATCC **CCL‐2**) and Vero cells (ATCC **CRL‐1587**) were cultured in Dulbecco's modified Eagle medium (DMEM) containing 10% fetal calf serum (FCS) as described in Reference 41. HSV1 propagation, titration and kinetics were done as described previously.^41^, ^42^ The strain HSV1(17^+^)lox (kindly provided by Beate Sodeik) was used as PCR template and for BAC mutagenesis. Plasmid transfection^12^ was performed using Effectene Transfection Reagent (Qiagen) while BAC transfection^42^ was done using Lipofectamine 2000 (Invitrogen).

### 4.2 NEX‐TRAP assay

The NEX‐TRAP was performed as reported.^12^, ^28^ In detail, HeLa cells were cotransfected with two plasmids, one encoding pCR3‐N‐HA‐UL10/gM‐FKBP and a second one encoding an EYFP‐NLS‐FRB‐Protein X. Following 20 hours of transfection, 50 μM Anisomycin was added to the cells. Following incubation for 15 minutes, Rapamycin was added at a concentration of 150 ng/mL. After incubation in presence of both Anisomycin and Rapamycin continued for 2 hours, the cells were processed for indirect immunofluorescence as described below. The plasmids encoding the tegument proteins were described before.^28^ Several plasmids carried partial sequences encoding truncated tegument proteins. These include the construct UL36a: nucleotides (nct) 1‐3000; UL36b: 3001‐6000; UL37d11: nct 34‐3372; UL46a: nct 1‐1008; UL46b: nct 1009‐2157.^28^

### 4.3 Leptomycin B assay for CRM1/Xpo1‐specific nuclear export

To analyze for CRM1/Xpo1‐specific nuclear export of tegument proteins, HeLa cells were transfected for 16 hours, then incubated with 50 ng/mL Leptomycin B for 3 hours, and subsequently treated with 50 μM Anisomycin for 1 hour in continued presence of Leptomycin B. Following indirect immunofluorescence, the subcellular distribution of myc‐tagged tegument proteins was quantified by classifying 100 cells per sample in a blinded experiment. Three different localization patterns were distinguished due to the highest signal strength.

### 4.4 Indirect immunofluorescence analysis

Transfected or infected HeLa or Vero cells were grown on sterile glass coverslips, fixed with 2% formaldehyde in PBS (15 minutes) and permeabilized with 0.5% Triton X‐100 (5 minutes, 4°C). Upon infection, binding of antibodies to the HSV1 Fc‐receptor‐like proteins gE or gI was blocked with human IgG in PBS (0.2 mg/mL) for at least 3 hours at room temperature.^41^ The NEX‐TRAP cargo protein was detected based on its fluorescent tag EYFP, the gM‐FKBP protein was detected using polyclonal rabbit anti‐gM antibodies (1:5000 in PBS) kindly provided by Thomas Mettenleiter. Anti‐ICP0 antibodies were purchased from Santa Cruz Biotechnology, anti‐VP16 (LP1), and anti‐VP5 (8F5) monoclonal antibodies were kindly provided by Gill Elliott, and Jay Brown, respectively. Monoclonal anti‐myc (9E10) antibodies were a gift of Jens von Einem. Goat anti‐mouse antibodies coupled to Alexa555 (subtype IgG1 specific), Alexa 488 (subtype IgG3 specific) or Alexa633 (Invitrogen) or goat anti‐rabbit antibodies coupled to Alexa555 or Alexa594 (Invitrogen) were used as secondary reagents. Nuclei were stained by Dapi. Cells were examined using the Leica TCS SP5 or a Zeiss confocal laser scanning microscope, and recorded using the Leica Application Suite AF6000 or the ZEN Lite Software. Brightness and contrast of images were adjusted using Adobe Photoshop or Corel Photo‐Paint. The scale of images is indicated by bars (10 μm).

### 4.5 BAC mutagenesis

HSV1 NES mutant and wild‐type strains were engineered using the BAC pHSV1(17^+^)lox and a modified version of the *galK*‐based BAC recombineering.^42^, ^43^ First, the UL48 locus encoding pUL48/VP16 was replaced by a *galK*‐kan cassette, which was amplified using the pGPS‐*galK*‐kan plasmid and the primers UL48/gk (Table 4) equipped with 50 bp homologies flanking the replaced region. Subsequently, this cassette was substituted with a fragment carrying UL48 where a NES mutation was inserted or with a wild‐type fragment. The mutant fragment was amplified by PCR using a plasmid as template carrying a wild‐type allele or the UL48 NES mutant previously generated by site‐directed mutagenesis. Both PCR fragments contained homologies to upstream and downstream sequences of the inserted *galK*‐kan cassette. Correct BAC sequences were confirmed by restriction pattern analysis and DNA sequencing.

**Table 4:** Primers used for BAC mutagenesis

UL48 seq	GCCAACTGACGCCAGCTCTC
UL48‐H5/gK	*ACGATCGCATCAAAAGCCCGATATCGTCTTTCCCGTATCAACCCCACCCA* CCTGTTGACAATTAATCATCGGCA
UL48‐H3/gK	*GCCGCATCATCTGCTCGGCGTACGCGGCCCATAGGATCTCGCGGGCCAAA* GCCAGTGTTACAACCAATTAACC
UL48‐H5/wt	*ACGATCGCATCAAAAGCCCGATATCGTCTTTCCCGTATCAACCCCACCCA* ATGGACCTCTTGGTCGACGAGCTG
UL48‐H3/wt	*GCCGCATCATCTGCTCGGCGTACGCGGCCCATAGGATCTCGCGGGCCAAA* AATAGATACAAATGCAAAAACAAA
UL48‐H3/NES	*GCCGCATCATCTGCTCGGCGTACGCGGCCCATAGGATCTCGCGGGCCAAA* **AATAGATACAAATGCAAAAACAAA**

Normal font: UL48 sequence; italic: flanking homologous regions; bold font: mutant sequence

### 4.6 Modeling of CRM1‐NES chimera

Structural representations were generated with UCSF Chimera (Petterson et al: UCSF Chimera—a visualization system for exploratory research and analysis). The structure of the pUL48 NES was obtained based on the structure of the Snurportin 1 NES bound to CRM1 (PDB ID 3NBY^30^). The model was subsequently energetically minimized using the AMBER ff14SB force field in Chimera.

### 4.7 Bioinformatic prediction of NESs

To predict NESs within herpesviral tegument proteins, several bioinformatic tools were applied including the NetNES (www.cbs.dtu.dk/services/NetNES 27), the prediction based on Bogerd et al^26^ called NES pattern, and the LocNES (http://prodata.swmed.edu/LocNES/LocNES.php 23). For systematic NES predictions in EBV proteins, sequences were obtained from Genbank (NCBI genome NC_007605.1^44^).

## CONFLICTS OF INTEREST

The authors have no conflict of interest to declare.

### Author contributions

CF, VR, DL, CCF, ZR, SMB conceived and designed the experiments. CF, VR, DL, JW, EA, JL performed the experiments. CF, VR, DL, JW, EA, JL, SMB analyzed the data. CCF, ZR, SMB contributed reagents/materials/analysis tools. CF, DL, SMB wrote the paper.

The Editorial Process File is available in the online version of this article.

### Editorial Process File

The Editorial Process File is available in the online version of this article.

## Supporting information


**Editorial Process**

**Figure S1** Validation of the subcellular localization of tegument proteins in absence and presence of Leptomycin B. HeLa cells were transfected with plasmids encoding myc‐tagged HSV1 tegument proteins. Protein synthesis was allowed for 16 hours, subsequently cells were treated with Leptomycin B for 3 hours or left untreated. In continuous presence of Leptomycin B the protein synthesis inhibitor Anisomycin was added for 1 hour. The myc‐tagged proteins were detected by an anti‐myc antibody. In 100 cells per sample, relative signal strengths in the nuclear and cytoplasmic region were evaluated by immunofluorescence analysis in a blinded manner. The signal was classified to be either predominantly cytoplasmic (N < C), predominantly nuclear (N > C), or equal in both compartments (N = C). A, Exemplary images showing the three classes of protein distribution (red) in cells transfected with pUL14, pUS3 or pUL7 expression plasmids, respectively. Nuclei were counterstained with Dapi (blue). The bar corresponds to 10 μm. B, Quantitative presentation of the localization of HSV1 proteins in absence (top panel) or presence (bottom panel) of Leptomycin B in a total of 100 cells per sample.
**Figure S2** NEX‐TRAP negative EBV tegument proteins. EBV tegument proteins pBBRF2 (pUL7), pBBLF1 (pUL11), pBGLF2 (pUL13) and pBLLF3 (pUL50) were subjected to NEX‐TRAP analysis to determine their nuclear export activity. After co‐transfection of the gM‐FKBP plasmid and the EYFP‐NLS‐FRB expression plasmid encoding one of the EBV proteins into HeLa cells, cells were incubated with anisomycin/rapamycin and protein localization was visualized by indirect immunofluorescence using rabbit anti‐gM antibody. Nuclei were counterstained with DAPI. With fluorescence microscopy, EYFP‐tagged proteins were visualized directly and gM by a secondary fluorophore‐tagged antibody. The bar corresponds to 10 μm.
**Table S1** Evidence for disordered and folded structures of NES regions. To determine the disordered and folded structures of the NESs identified in a group of exported HSV1 tegument proteins, the in silico tool PredictProtein was applied to the full‐length proteins. For pUL21 and pUL37, a crystal structure is available that was additionally used to determine the secondary structure of the proteins.
