# Supplementary material for: Comprehensive analysis of nuclear export of herpes simplex virus type 1 tegument proteins and their Epstein‐Barr virus orthologs
Source: Traffic. 2019 Jan 11;20(2):152–67. doi: 10.1111/tra.12627 (PMC6590417; doi:10.1111/tra.12627)
Supplement: Supplementary file 2 — Figure S1 Validation of the subcellular localization of tegument proteins in absence and presence of Leptomycin B. HeLa cells were transfected with plasmids encoding myc‐tagged HSV1 tegument proteins. Protein synthesis was allowed for 16 hours, subsequently cells were treated with Leptomycin B for 3 hours or left untreated. In continuous presence of Leptomycin B the protein synthesis inhibitor Anisomycin was added for 1 hour. The myc‐tagged proteins were detected by an anti‐myc antibody. In 100 cells per sample, relative signal strengths in the nuclear and cytoplasmic region were evaluated by immunofluorescence analysis in a blinded manner. The signal was classified to be either predominantly cytoplasmic (N < C), predominantly nuclear (N > C), or equal in both compartments (N = C). A, Exemplary images showing the three classes of protein distribution (red) in cells transfected with pUL14, pUS3 or pUL7 expression plasmids, respectively. Nuclei were counterstained with Dapi (blue). The bar corresponds to 10 μm. B, Quantitative presentation of the localization of HSV1 proteins in absence (top panel) or presence (bottom panel) of Leptomycin B in a total of 100 cells per sample. Figure S2 NEX‐TRAP negative EBV tegument proteins. EBV tegument proteins pBBRF2 (pUL7), pBBLF1 (pUL11), pBGLF2 (pUL13) and pBLLF3 (pUL50) were subjected to NEX‐TRAP analysis to determine their nuclear export activity. After co‐transfection of the gM‐FKBP plasmid and the EYFP‐NLS‐FRB expression plasmid encoding one of the EBV proteins into HeLa cells, cells were incubated with anisomycin/rapamycin and protein localization was visualized by indirect immunofluorescence using rabbit anti‐gM antibody. Nuclei were counterstained with DAPI. With fluorescence microscopy, EYFP‐tagged proteins were visualized directly and gM by a secondary fluorophore‐tagged antibody. The bar corresponds to 10 μm. Table S1 Evidence for disordered and folded structures of NES regions. To determine the disordered and folded [file TRA-20-152-s001.pdf]

Comprehensive analysis of nuclear export of herpes simplex virus type 1 tegument proteins and their Epstein-Barr virus orthologs

Christina Funk[1,¶], Verena Raschbichler[2,¶], Diana Lieber[2,3,¶], Jens Wetschky[1], Eileen Arnold[4], Jacqueline Leimser[4], Michael Biggel[1], Caroline C. Friedel[5], Zsolt Ruzsics[6], Susanne M. Bailer[1,2,4,*]

[1] Fraunhofer-Institut für Grenzflächen- und Bioverfahrenstechnik IGB, Stuttgart, Germany
[2] Max von Pettenkofer-Institute, Ludwig-Maximilians-University Munich, München, Germany
[3] Institute of Virology, Ulm University Medical Center, Ulm, Germany
[4] Biological Interfacial Engineering, University of Stuttgart, Stuttgart, Germany
[5] Institute for Informatics, Ludwig-Maximilians-University Munich, München, Germany
[6] Institute of Virology, Medical Center – University of Freiburg, Medical Faculty, University of Freiburg, Freiburg, Germany

[¶] These authors should be considered joint first authors
[*] Corresponding author

Adress:
Prof. Dr. Susanne M. Bailer
Institut für Grenzflächenverfahrenstechnik und Plasmatechnologie IGVP
der Universität Stuttgart
Nobelstraße 12
70569 Stuttgart
Germany
Email: Susanne.Bailer@igvp.uni-stuttgart.de

Supplemental Materials

Suppl. Table 1: Evidence for disordered and folded structures of NES regions. To determine the disordered and folded structures of the NESes identified in a group of exported HSV1 tegument proteins, the *in silico* tool PredictProtein was applied to the full length proteins. For pUL21 and pUL37, a crystal structure is available that was additionally used to determine the secondary structure of the proteins.

	**Predicted and / or validated NES**	**Secondary structure**		**Source**
		**Folded (Helix)**	**Disordered**	
**pUL4**	^{172}**PTADLLVEVLREIQL**^{186}	§ 175-184	* 172-174; 179; 182; 184-186	UniProtKB - P10188 (NP04_HHV11)
**pUL13**	^{492}**LPPELKPLLVLVSRL**^{506}	§ 494 – 506	* 492-498	UniProtKB - P04290 (UL13_HHV11)
**pUL21**	^{476}**ATHTARLTGVTSLVL**^{490}	+ 476-483; 487-490<br>§ 476; 477 – 478; 486-490	* 484	UniProtKB - P10205 (TEG4_HHV11)
**pUL37**	^{258}**ATLTPLTRALFTLAL**^{272}	# 262-272	* 258-261	UniProtKB - P10221 (ITP_HHV11)
**pUL37** (score 0.620)	^{763}LEDAIVLLRLHMRTL^{777}	§ 763-777	* 764-765	
**pUL47**	^{282}**QAMSFLADAVVRLAI**^{296}	§ 283-296	* 282	UniProtKB - P10231 (TEG5_HHV11)
**pUL47** (score 0.247)	^{651}**PRVRVVDIMSQFRKL**^{665}	§ 321-325	* 314-325; 327-328	
**pUL48**	^{220}**ARLARVLFLHLYLFL**^{234}	§ 220-234		UniProtKB - P04486 (VP16_HHV1F)
Bold sequence: experimentally tested NES. Numbers in column 2, 3, and 4 indicate amino acid residues.<br>§ PredictProtein REPROFSec<br>* PredictProtein PROFbval<br># PDB 5VYL - P10221 (ITP_HHV11)<br>+ PDB 5ED7 - P10205 (TEG4_HHV11)				

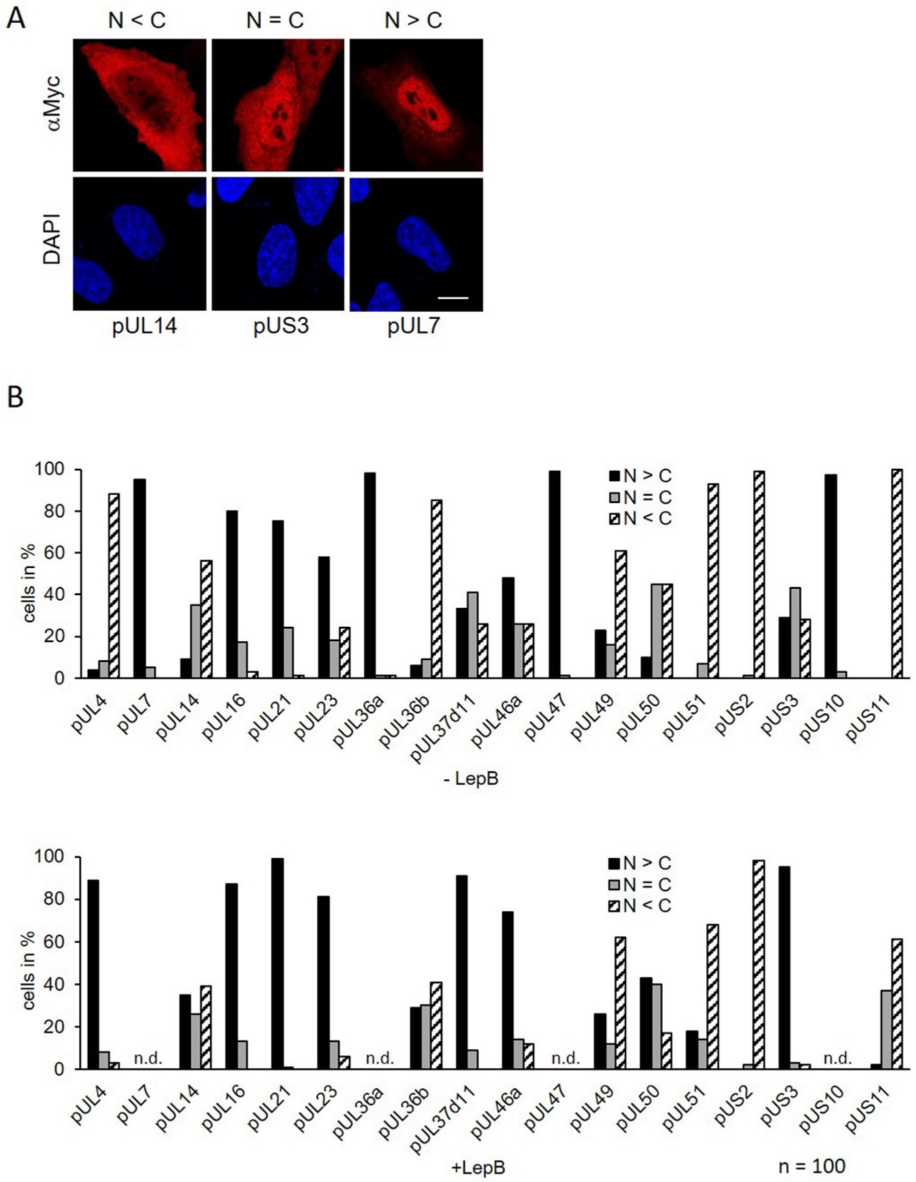

S1 Figure: Validation of the subcellular localization of tegument proteins in absence and presence of Leptomycin B.

S1 Figure: Validation of the subcellular localization of tegument proteins in absence and presence of Leptomycin B. HeLa cells were transfected with plasmids encoding myc-tagged HSV1 tegument proteins. Protein synthesis was allowed for 16 h, subsequently cells were treated with Leptomycin B for 3 h or left untreated. In continuous presence of Leptomycin B the protein synthesis inhibitor Anisomycin was added for 1 h. The myc-tagged proteins were detected by an anti-myc antibody. In 100 cells per sample, relative signal strengths in the nuclear and cytoplasmic region were evaluated by immunofluorescence analysis in a blinded manner. The signal was classified to be either predominantly cytoplasmic (N < C), predominantly nuclear (N > C), or equal in both compartments (N = C). (A) Exemplary images showing the three classes of protein distribution (red) in cells transfected with pUL14, pUS3, or pUL7 expression plasmids, respectively. Nuclei were counterstained with Dapi (blue). The bar corresponds to 10 µm. (B) Quantitative presentation of the localization of HSV1 proteins in absence (top panel) or presence (bottom panel) of Leptomycin B in a total of 100 cells per sample.

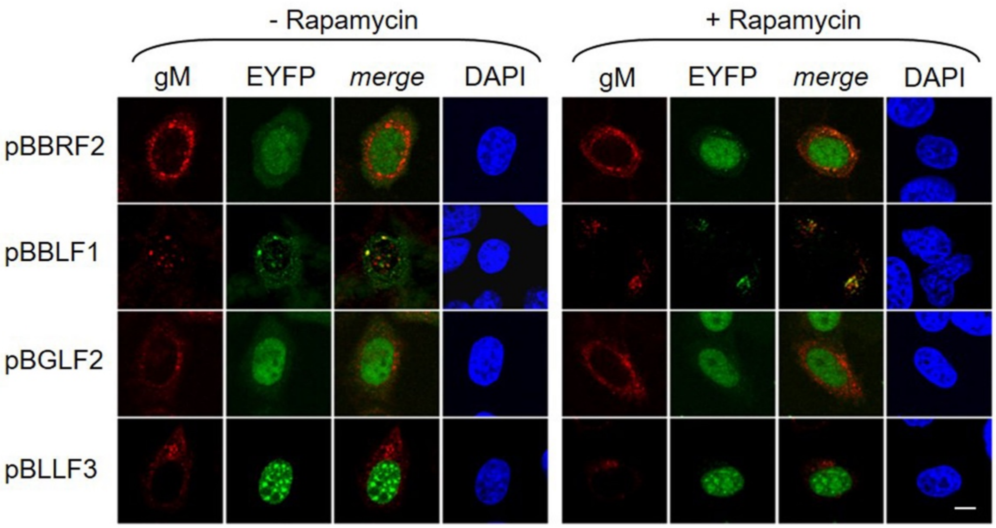

S2 Figure: NEX-TRAP negative EBV tegument proteins.

S2 Figure: NEX-TRAP negative EBV tegument proteins. EBV tegument proteins pBBRF2 (pUL7), pBBLF1 (pUL11), pBGLF2 (pUL13), and pBLLF3 (pUL50) were subjected to NEX-TRAP analysis to determine their nuclear export activity. After co-transfection of the gM-FKBP plasmid and the EYFP-NLS-FRB expression plasmid encoding one of the EBV proteins into HeLa cells, cells were incubated with Anisomycin/Rapamycin and protein localization was visualized by indirect immunofluorescence using rabbit anti-gM antibody. Nuclei were counterstained with DAPI. With fluorescence microscopy, EYFP-tagged proteins were visualized directly and gM by a secondary fluorophore-tagged antibody. The bar corresponds to 10 µm.
