# Supplementary material for: Comprehensive analysis of nuclear export of herpes simplex virus type 1 tegument proteins and their Epstein‐Barr virus orthologs
Source: Traffic. 2019 Jan 11;20(2):152–67. doi: 10.1111/tra.12627 (PMC6590417; doi:10.1111/tra.12627)
Supplement: Supplementary file 1 — Editorial Process [file TRA-20-152-s002.pdf]

Title: **Comprehensive analysis of nuclear export of herpes simplex virus type 1 tegument proteins and their Epstein-Barr virus orthologs**

Authors: Christina Funk, Verena Raschbichler, Diana Lieber, Jens Wetschky, Eileen Arnold, Jacqueline Leimser, Michael Biggel, Caroline C. Friedel, Zsolt Ruzsics and Susanne M. Bailer

Article Type: Original research

Monitoring Editor Sharon Tooze
Date Submitted 10 May 2018
Date for Decision 1 19 June 2018
Date Resubmitted 17 September 2018
Date for Decision 2 12 October 2018
Accepted 29 November 2018

Decision and Reviews

Dear Dr. Bailer,

Thank you for submitting your manuscript “Nuclear export of herpes simplex virus type 1 tegument proteins and their Epstein-Barr virus orthologs” to Traffic. I asked two colleagues who are experts in the field to review the paper and their verbatim comments are appended below. The referees share the view that the work presented in this paper is of a high caliber and will be of interest to the readers of Traffic. However, both referees raise concerns about the presentation and discussion that will require revisions. Since this will not require the inclusion of additional data, I believe you will be able to address the referees concerns fairly quickly and I look forward to receiving your revised paper in the near future. To expedite handling when you resubmit please be sure to include a response outlining how you have addressed each of the referees’ concerns as well as a version of the text in which the revisions have been marked.

Sincerely,

Sharon A. Tooze, Ph.D.
Co-Editor

Referee's Comments to the Authors

Referee: 1

Comments to the Author
The group of Susanne Bailer has submitted a manuscript describing an in-depth, systematic functional characterization of the activity of predicted nuclear export signals (NES) in HSV-1 and EBV proteins. For this, they used their NEX-TRAP system (nuclear export trapped by rapamycin; Raschbichler et al. 2012, Traffic) which relies on fluorescence microscopy and rapamycin-induced interaction of a protein-of-interest with the integral membrane protein gM at the trans-Golgi-network. Using this assay, the authors could validate an NES activity in 9 of 22 bioinformatically predicted HSV-1 and in 2 of 6 predicted EBV tegument proteins.
The authors' work clearly indicates that the prediction algorithms NetNES, LocNES and NES pattern help to generate

hypotheses, but that an unambiguous identification of an NES activity in a protein of interest requires experimental validation. The authors demonstrate that their NEX-TRAP system is a good tool to do so although it has its limitations too, which the authors addressed in detail in their discussion. The author's work therefore advance the field on understanding nuclear export signals in viral and host proteins, and in particular provide an important compilation on the mechanisms to control the subcellular localization of the tegument proteins of HSV-1 and EBV.
The experimental analyses have been well designed and executed; however, the paper is cumbersome to read, and one gets easily lost among the different abbreviations, tables and convoluted sentences. Many sentences would benefit from plain shortening and deleting redundancies and superfluous words. While this reviewer understands that a substantial amount of work went into compiling these data, it should not require a substantial amount of own note taking to appreciate the results. The authors need to take one step back for a global perspective and summarized their data for HSV-1 and EBV in one table each; otherwise, their message will not reach their readers.

Specific comments:
1. Table 1 deals with tegument proteins of different herpesviruses not just those of HSV-1; thus, the subheadings of the table are inconsistent. The second column contains incomplete information, and furthermore could be easily fused with the first column. It is unclear why for some proteins the enzymatic activities and alternative names have been listed, but for others not. Along the same lines, why did the authors list the molecular weights for the HSV-1 proteins but not for the other viruses? Please revise accordingly.
2. Results: The result section often contains too many experimental details, and also too many redundancies that severely impair the flow of reading and understanding. This reviewer strongly supports an in-detail description of all experiments, but the authors should move and/or limit details to the Materials and Methods and to the Figure legend sections. An example for many redundancies is for example the number of times it is written that the activity of export factor CRM1 is inhibited by leptomycin B.
3. The results of the Figure 2 and the S1 Figure should be added to Table 5 which currently only lists the bioinformatics predictions. Table 5 should have the NES that were tested experimentally underlined. According to this, Table 3 could then be deleted, since all NES had been mutated in the same manner (LxxxAxxAxA). Furthermore, Table 5 should include the subcellular localization in response to the leptomycin treatment, and the results of all bioinformatic algorithms, not just the LocNES. The second column of the current Figure 5 should be deleted. It is incomplete and redundant with an identical column in Figure 1. The protein positions listed in the present column 3 could be integrated with column 4.
4. The presentation of the results for the EBV proteins should be revised as requested above for the HSV-1 proteins. Table 4 is redundant too, and should be deleted. Table 6 does not need to list the HSV-1 orthologs; this has been done already in Table 1.
5. Discussion, page 6, second column: There seems to be a mistake; this should read "… masking their potential nuclear export activity, thereby masking a potential nuclear export activity".

Referee: 2

Comments to the Author
The authors systematically tested HSV1 and EBV tegument proteins using the NEX-TRAP assay to identify proteins that participate in nuclear export. They found that 9 of the 22 HSV1 and 2 of 6 EBV tegument proteins tested have nuclear export activities. They used bioinformatics tools to identify NESs in six of the tegument proteins. The experimental results identifying tegument proteins that are exported are solid. The studies identifying and testing NESs are less conclusive. Overall, the study is important, advance knowledge on cell biology of viruses and is suitable for publication in Traffic.

Major concerns:

1. In this study, the authors seem to make a comparison between bioinformatics tools and their NEX TRAP assay, and the description of this comparison is quite confusing. In silico NES prediction methods are developed to search for potential NES-containing proteins and potential NES sequences that could then be subjected to experimental testing or validation. In silico prediction is not meant to replace experiments. To identify potential CRM1 cargos, researchers usually apply bioinformatics tools to proteins of interest, retrieve the highest predicted score for each protein and generate a ranked list, which is useful for selecting a manageable number of candidates to be tested experimentally. In fact, this seems what the authors did for EBV tegument proteins in Table 6. In silico screening is not necessary if the plan was to test nuclear export of ALL HSV1 tegument proteins by NEX TRAP assay. In silico analysis is then really more suitable to identify potential NES sequences in the proteins that are found by experiments to be exported. It does not make much sense to compare LocNES with NEX TRAP assay. They serve different purposes.

2. A potential NES sequence merely indicates potential nuclear export activity. There are many potential NESs in the proteome, probably as many as one in every protein, given the many different hydrophobic patterns that have been found in active NESs (Kosugi et al PLOS Computational Biology 2014 and Fung et al eLife 2015). This high degree of variability in sequence is in turn explained by the many different conformations an NES peptide can adopt when binding CRM1. Amongst the many potential NESs, most do not confer nuclear export activity.
LocNES and other prediction tools score a potential NES for how likely it will confer nuclear export activity. There are two ways potential NESs may fail to mediate nuclear export. 1) Not all sequences that match NES consensus patterns can bind CRM1 (Xu et al MBoC 2012) and the field is still trying to figure out how to distinguish sequences that will bind from those that do not. 2) Furthermore, most potential NES sequences, because of the presence of multiple hydrophobic amino acids, are actually found in the interior of folded protein domains. This means that even though many sequences look like NESs, they are buried and therefore not accessible to bind CRM1. LocNES and other bioinformatics tools such as NESsential (Fu et al Nucleic Acids Res. 2011) predict accessibility of the potential NES within the protein, and use that accessibility and other factors to score the NES. Considering all potential NESs is meaningless. One must take into account the score that assesses the likelihood that a potential NES sequence will confer nuclear export activity.
Perhaps the authors could explain in more detail how they found NES candidates within the HSV1 tegument proteins that tested positive in their NEX TRAP assay. For example, comparing different bioinformatics tools and discussing which facilitates the most in pinpointing NES within HSV1 tegument proteins.

3. In Figure 5, the authors compare different NES predictions and NEX-TRAP activity of HSV1 and EBV tegument proteins. I suggest that the authors select top-ranked prediction results, instead of all of them, and re-illustrate by Venn diagram. Using score- or probability-based predictors, the authors should be able to sort the scores of the candidates and apply certain threshold to acquire top-ranked candidates. For example, Prieto et al. used first quantile (Q1) to select higher-score candidates in their recent study (Prieto et al Scientific Reports 2016). Users of bioinformatics tools usually select candidates with high scores, rather than accepting all candidates unconditionally. For EBV tegument proteins, this will generate a list of candidates that are ranked high by different predictors. This information will be useful for future validation.

4. The NES consensus sequence from Bogerd et al. Molecular and cell biology 1996 used in this study is ancient. NES patterns have expanded significantly in the last 10 years (Kosugi et al Traffic 2008, Kosugi et al PLOS Computational Biology 2014, Fung et al eLife 2015, and Liku et al BMC Bioinformatics 2018). If the authors want to find a profile-based prediction method, NESmapper is a more appropriate updated tool (Kosugi et al PLOS Computational Biology 2014).

5. The authors did not analyze the locations of the putative NESs that they mutated in Fig 3B, within their full-length protein sequences. With the exception of the sequences in pUL4, pUL13 and pUL37, the other sequences scored really low and may not be accessible within the full-length proteins. Curiously the highest scoring NES sequence in pUL37 was not picked to be tested. Are the NES sequences that were tested likely to be in long loops or disordered regions of the proteins? Otherwise why would they be expected to be accessible to CRM1?

6. Mutations that abolish nuclear export do not necessarily mark nuclear export signals. For example, mutating hydrophobic residues often disrupt the folds of proteins and thus disrupt many different functions of the proteins. Mutations may also disrupt binding to partner proteins with active NESs, and thus abolish critical indirect interactions that prevent nuclear export. Here are a few examples of misidentified NESs: Hantschel et al Mol Cell 2005, Ederle et al Sci Rep. 2018 and Pinarbasi et al Sci Rep 2018.

7. Do the NES sequences that the authors tested bind CRM1, even as peptides? CRM1-binding is a key and necessary feature of NESs. Can these potential NES peptides direct nuclear export of a heterologous reporter protein?

Minor comments:

1. The last sentence of the 3rd paragraph in Introduction: “Therefore, the export activity of a protein needs to be determined empirically, in vivo and in the context of the folded protein, and predicted NESs require experimental confirmation.” Did the authors mean “empirically” or “experimentally”?

2. In the 2nd paragraph of the Results section of Validation of the nuclear export activity of HSV1 tegument proteins, the authors first said that 5 proteins (pUL4, pUL11, pUL13, pUL21, pUL37 showed positive nuclear export in NEX-TRAP

and showed LMB sensitivity. It is confusing why they then omitted pUL4 from this list and say that only the other four proteins are exported by CRM1. Why did they drop pUL4 from this list? They didn't show pUL4 in Fig 2B but the data for pUL4 in Fig S1B looks pretty good.

Author Rebuttal

Dear Dr. Tooze,
please find enclosed our manuscript "Comprehensive analysis of nuclear export of herpes simplex virus type 1 tegument proteins and their Epstein-Barr virus orthologs" by Funk, Raschbichler, Lieber et al., to be considered for publication in Traffic. As detailed in the rebuttal, we have carefully revised the manuscript. We hope we adressed all critical points of the referees.
Thank you very much for your consideration.
Sincerely,
Susanne Bailer

Rebuttal to Referee: 1

Comments to the Author
The group of Susanne Bailer has submitted a manuscript describing an in-depth, systematic functional characterization of the activity of predicted nuclear export signals (NES) in HSV-1 and EBV proteins. For this, they used their NEX-TRAP system (nuclear export trapped by rapamycin; Raschbichler et al. 2012, Traffic) which relies on fluorescence microscopy and rapamycin-induced interaction of a protein-of-interest with the integral membrane protein gM at the trans-Golgi-network. Using this assay, the authors could validate an NES activity in 9 of 22 bioinformatically predicted HSV-1 and in 2 of 6 predicted EBV tegument proteins.
The authors' work clearly indicates that the prediction algorithms NetNES, LocNES and NES pattern help to generate hypotheses, but that an unambiguous identification of an NES activity in a protein of interest requires experimental validation. The authors demonstrate that their NEX-TRAP system is a good tool to do so although it has its limitations too, which the authors addressed in detail in their discussion. The author's work therefore advance the field on understanding nuclear export signals in viral and host proteins, and in particular provide an important compilation on the mechanisms to control the subcellular localization of the tegument proteins of HSV-1 and EBV.
The experimental analyses have been well designed and executed; however, the paper is cumbersome to read, and one gets easily lost among the different abbreviations, tables and convoluted sentences. Many sentences would benefit from plain shortening and deleting redundancies and superfluous words. While this reviewer understands that a substantial amount of work went into compiling these data, it should not require a substantial amount of own note taking to appreciate the results. The authors need to take one step back for a global perspective and summarized their data for HSV-1 and EBV in one table each; otherwise, their message will not reach their readers.

> First, we would like to thank this reviewer for the careful review and all comments and suggestions. Based on those, we believe that we were able to generate an improved manuscript that is much easier to read and transports the message to the reader interested in nuclear export with a particular focus on export of viral proteins.

Specific comments:
1. Table 1 deals with tegument proteins of different herpesviruses not just those of HSV-1; thus, the subheadings of the table are inconsistent. The second column contains incomplete information, and furthermore could be easily fused with the first column. It is unclear why for some proteins the enzymatic activities and alternative names have been listed, but for others not. Along the same lines, why did the authors list the molecular weights for the HSV-1 proteins but not for the other viruses? Please revise accordingly.

> We have corrected the subheading of Table 1.
> The second column indicates activities and alternative names given to some but not all tegument proteins. Therefore, in some cases, there are no alternative names.
> The molecular weights of herpesviruses other than HSV1 have been inserted in Table 1.

2. Results: The result section often contains too many experimental details, and also too many redundancies that severely impair the flow of reading and understanding. This reviewer strongly supports an in-detail description of all experiments, but the authors should move and/or limit details to the Materials and Methods and to the Figure legend sections. An example for many redundancies is for example the number of times it is written that the activity of export factor CRM1 is inhibited by leptomycin B.

> We have revised the manuscript as indicated in the track recorded file, with a specific focus on the results section.

3. The results of the Figure 2 and the S1 Figure should be added to Table 5 which currently only lists the bioinformatics predictions. Table 5 should have the NES that were tested experimentally underlined. According to this, Table 3 could then be deleted, since all NES had been mutated in the same manner (LxxxAxxAxA). Furthermore, Table 5 should include the subcellular localization in response to the leptomycin treatment, and the results of all bioinformatic algorithms, not just the LocNES. The second column of the current Figure 5 should be deleted. It is incomplete and redundant with an identical column in Figure 1. The protein positions listed in the present column 3 could be integrated with column

> We have summarized the data for HSV1 and EBV in one table each (Table 2 and 3, respectively). In Table 2, the experimentally tested NES are in bold, the consensus hydrophobic residues are underlined. As suggested, we have added the results of Figure 2 and S1 Figure to Table 2. Previous Table 3 was deleted. Furthermore, we have added the subcellular localization in response to the leptomycin B treatment and all bioinformatics results. The second column was deleted. The protein positions listed in previous column 3 are integrated in previous column 4.

4. The presentation of the results for the EBV proteins should be revised as requested above for the HSV-1 proteins. Table 4 is redundant too, and should be deleted. Table 6 does not need to list the HSV-1 orthologs; this has been done already in Table 1.

> The results for the EBV proteins (Table 3) were revised as described for the HSV1 proteins (Table 2). Previous Table 4 is deleted.

5. Discussion, page 6, second column: There seems to be a mistake; this should read "… masking their potential nuclear export activity, thereby masking a potential nuclear export activity".

> The sentence has been altered to "The strong SV40-NLS may have led to a predominantly nuclear localization of the NES (282 - 296) fusion protein masking its potential nuclear export activity."

Rebuttal to Referee: 2

Comments to the Author
The authors systematically tested HSV1 and EBV tegument proteins using the NEX-TRAP assay to identify proteins that participate in nuclear export. They found that 9 of the 22 HSV1 and 2 of 6 EBV tegument proteins tested have nuclear export activities. They used bioinformatics tools to identify NESs in six of the tegument proteins. The experimental results identifying tegument proteins that are exported are solid. The studies identifying and testing NESs are less conclusive. Overall, the study is important, advance knowledge on cell biology of viruses and is suitable for publication in Traffic.

Major concerns:

1. In this study, the authors seem to make a comparison between bioinformatics tools and their NEX TRAP assay, and the description of this comparison is quite confusing. In silico NES prediction methods are developed to search for potential NES-containing proteins and potential NES sequences that could then be subjected to experimental testing or validation. In silico prediction is not meant to replace experiments. To identify potential CRM1 cargos, researchers usually apply bioinformatics tools to proteins of interest, retrieve the highest predicted score for each protein and generate a ranked list, which is useful for selecting a manageable number of candidates to be tested experimentally. In fact, this seems what the authors did for EBV tegument proteins in Table 6. In silico screening is not necessary if the plan was to test nuclear export of ALL HSV1 tegument proteins by NEX TRAP assay. In silico analysis is then really more suitable to identify potential NES sequences in the proteins that are found by experiments to be exported. It does not make much sense to compare LocNES with NEX TRAP assay. They serve different purposes.

> We would like to thank this reviewer for these important and helpful comments. We have addressed this by restructuring the results section of the manuscript. i) We describe the NEX-TRAP data and the confirmation of nuclear export by Leptomycin B treatment. ii) This experimental part is then followed by the in silico analysis to identify potential NES sequences that iii) are verified by mutational analysis using the NEX-TRAP. This also reflects the process used to analyse EBV tegument proteins. We have also described this approach in the discussion section.

2. A potential NES sequence merely indicates potential nuclear export activity. There are many potential NESs in the proteome, probably as many as one in every protein, given the many different hydrophobic patterns that have been found in active NESs (Kosugi et al PLOS Computational Biology 2014 and Fung et al eLife 2015). This high degree of variability in sequence is in turn explained by the many different conformations an NES peptide can adopt when binding CRM1. Amongst the many potential NESs, most do not confer nuclear export activity.
LocNES and other prediction tools score a potential NES for how likely it will confer nuclear export activity. There are two ways potential NESs may fail to mediate nuclear export. 1) Not all sequences that match NES consensus patterns can bind CRM1 (Xu et al MBoC 2012) and the field is still trying to figure out how to distinguish sequences that will bind from those that do not. 2) Furthermore, most potential NES sequences, because of the presence of multiple hydrophobic amino acids, are actually found in the interior of folded protein domains. This means that even though many sequences look like NESs, they are buried and therefore not accessible to bind CRM1. LocNES and other bioinformatics tools such as NESsential (Fu et al Nucleic Acids Res. 2011) predict accessibility of the potential NES within the protein, and use that accessibility and other factors to score the NES. Considering all potential NESs is meaningless. One must take into account the score that assesses the likelihood that a potential NES sequence will confer nuclear export activity. Perhaps the authors could explain in more detail how they found NES candidates within the HSV1 tegument proteins that tested positive in their NEX TRAP assay. For example, comparing different bioinformatics tools and discussing which facilitates the most in pinpointing NES within HSV1 tegument proteins.

> We fully agree with the reviewer that most potential NESes do not confer nuclear export. We have extended our discussion and proposed the way we identified the NESes of viral tegument proteins, by i) determining nuclear export activity of a protein using the NEX-TRAP, and/or taking another experimental approach; by ii) using at least two different in silico tools; by iii) mutating the predicted NES in the context of the full length protein and testing it in an experimental setting; and by iv) performing further experiments, as suggested in comment #7 (binding of the NES peptide to CMR1, or binding of the wildtype and mutant protein to CRM1).

> As pointed out in comment #1, we have restructured the results section of the manuscript to reflect the overall approach we took.

3. In Figure 5, the authors compare different NES predictions and NEX-TRAP activity of HSV1 and EBV tegument proteins. I suggest that the authors select top-ranked prediction results, instead of all of them, and re-illustrate by Venn diagram. Using score- or probability-based predictors, the authors should be able to sort the scores of the candidates and apply certain threshold to acquire top-ranked candidates. For example, Prieto et al. used first quantile (Q1) to select higher-score candidates in their recent study (Prieto et al Scientific Reports 2016). Users of bioinformatics tools usually select candidates with high scores, rather than accepting all candidates unconditionally. For EBV tegument proteins, this will generate a list of candidates that are ranked high by different predictors. This information will be useful for future validation.

> We have compared our experimental data (NEX-TRAP and LepB responsiveness) to the in silico data (based on Loc-NES, NES pattern and NetNES) in the new Table 2. The new composition readily shows that for several exported tegument proteins, Loc-NES predicted only one NES that was also identified by a second in silico approach and matched the experimentally identified NES (pUL4, pUL13, and pUL21). In other cases (pUL37, pUL47, and pUL48), Loc-NES predicted several potential NESes with one matching the prediction of a second in silico tool that rarely ranked highest.

> Table 3 (previous Table 6) is fully revised to include all LocNES predictions with their scores as well as the NEs pattern predictions. We think that this information provides the basis for future validations.

> Based on our data we suggest and discuss the following way to identify a functional NES, i) by determining nuclear export activity using the NEX-TRAP, and/or taking another experimental approach, ii) using at least two different in silico tools, iii) mutating the predicted NES and testing it in an experimental setting, and iv) performing further experiments like those suggested in comment #7.

4. The NES consensus sequence from Bogerd et al. Molecular and cell biology 1996 used in this study is ancient. NES patterns have expanded significantly in the last 10 years (Kosugi et al Traffic 2008, Kosugi et al PLOS Computational Biology 2014, Fung et al eLife 2015, and Liku et al BMC Bioinformatics 2018). If the authors want to find a profile-based prediction method, NESmapper is a more appropriate updated tool (Kosugi et al PLOS Computational Biology 2014).

> We agree with the reviewer that Bogerd et al., 1996 is a rather early publication. Still, the bioinformatics tool NES pattern that was developed based on the consensus sequence defined therein worked very well and identified a NES in almost all exported tegument proteins. For more recent bioininformatic prediction tools, we have added the new references (Kosugi et al Traffic 2008, Kosugi et al PLOS Computational Biology 2014, Fung et al eLife 2015, and Liku et al BMC Bioinformatics 2018) to the introduction, the results and discussion sections.

5. The authors did not analyze the locations of the putative NESs that they mutated in Fig 3B, within their full-length protein sequences. With the exception of the sequences in pUL4, pUL13 and pUL37, the other sequences scored really low and may not be accessible within the full-length proteins. Curiously the highest scoring NES sequence in pUL37 was not picked to be tested. Are the NES sequences that were tested likely to be in long loops or disordered regions of the proteins? Otherwise why would they be expected to be accessible to CRM1?

> For the exported HSV1 tegument proteins we identified the same NES using two different prediction tools, the NES pattern (or the NetNES) AND LocNES. In most cases, the overlapping and experimentally confirmed NES predicted by LocNES did not achieve the highest score. Most dramatically, for pUL21 only one NES was predicted by LocNES that overlapped with the NES pattern prediction but scored very low. Targeted mutagenesis of this NES abolished nuclear export of pUL21. While this needs further experimental validation as generally discussed in the revised manuscript, this strongly suggests that the score is not a good indicator for picking the most promising NES.

6. Mutations that abolish nuclear export do not necessarily mark nuclear export signals. For example, mutating hydrophobic residues often disrupt the folds of proteins and thus disrupt many different functions of the proteins. Mutations may also disrupt binding to partner proteins with active NESs, and thus abolish critical indirect interactions that prevent nuclear export. Here are a few examples of misidentified NESs: Hantschel et al Mol Cell 2005, Ederle et al Sci Rep. 2018 and Pinarbasi et al Sci Rep 2018.

> We are fully aware of these potential pitfalls for mutating NESes. We have extended the discussion and describe the potential limitations of NES mutagenesis in the discussion. We have also included the indicated references of misidentified NESes.

7. Do the NES sequences that the authors tested bind CRM1, even as peptides? CRM1-binding is a key and necessary feature of NESs. Can these potential NES peptides direct nuclear export of a heterologous reporter protein?

> We fully agree with the reviewer that binding of these peptides to CRM1 is an important feature of a functional NES and have included this requirement in the discussion section. We believe that this manuscript provides detailed systematic analysis of nuclear export of HSV1 and in part of EBV tegument proteins. Further efforts to support and characterize their mode of nuclear export and the functional impact on viral replication are however beyond the scope of this manuscript.

Minor comments:

1. The last sentence of the 3rd paragraph in Introduction: “Therefore, the export activity of a protein needs to be determined empirically, in vivo and in the context of the folded protein, and predicted NESs require experimental confirmation.” Did the authors mean “empirically” or “experimentally”?
> We have exchanged “empirically” with “experimentally”.

2. In the 2nd paragraph of the Results section of Validation of the nuclear export activity of HSV1 tegument proteins, the authors first said that 5 proteins (pUL4, pUL11, pUL13, pUL21, pUL37 showed positive nuclear export in NEX-TRAP and showed LMB sensitivity. It is confusing why they then omitted pUL4 from this list and say that only the other four proteins are exported by CRM1. Why did they drop pUL4 from this list? They didn’t show pUL4 in Fig 2B but the data for pUL4 in Fig S1B looks pretty good.
-
> Since nuclear export of pUL4 was characterized in a previous publication, we did not include it in Fig. 2B. We have however corrected the number of tegument proteins that are found to be actively exported in a CRM1-dependent manner from 4 to 5.

Decision and Reviews

Dear Dr. Bailer,

Thank you for submitting your revised manuscript "Nuclear export of herpes simplex virus type 1 tegument proteins and their Epstein-Barr virus orthologs" to Traffic. I asked the referees to read the revised paper and their verbatim comments are appended below. I agree with the referees that you have addressed most of the concerns raised previously. However, a few revisions are required before this paper can be accepted. Referee 1 identifies several points that need to be clarified. Referee 2 recommends including one additional analysis of the location and accessibility of the potential NES sequences. This request was in the original Referee report and seems entirely straightforward to do and include.

Although I cannot accept your manuscript for publication at this point, I believe that you will be able to address these remaining concerns and I look forward to receiving your revised manuscript. To expedite handling when you resubmit please be sure to include a response outlining how you have addressed each of the referees' concerns.

Sincerely,

Sharon A. Tooze, PhD
Co-Editor

Referee's Comments to the Authors

Referee: 1

Comments to the Author
The authors have done a very good job in considering the reviewer's comments on their study. They have taken care of most my comments, and the manuscript has improved considerably. The authors have decided against shortening some redundancies, but they seem to prefer their manuscript to remain like that. I have some few minor comments left.
1. At many (all?) places the tegument protein name pUL37 has been replaced by pUL37d11. I presume this is a spelling error which should be corrected. If not, please provide an explanation; is this a truncated version of pUL37?
2. In the discussion, the authors mention that they only covered experimentally only a N-terminal piece of the tegument protein pUL36 but nowhere provide Information how large this piece was. They should add this information to the summarizing table 2 (aa1-aaXXX).
3. I presume besides pUL36, all other constructs expressed full-length proteins. If not, the domains that have been used in the experiments should be indicated in tables 2 and 3.

Referee: 2

Comments to the Author
The authors did not address comment #5 of reviewer 2 -
"5. The authors did not analyze the locations of the putative NESs that they mutated in Fig 3B, within their full-length protein sequences. With the exception of the sequences in pUL4, pUL13 and pUL37, the other sequences scored really low and may not be accessible within the full-length proteins. Curiously the highest scoring NES sequence in pUL37 was not picked to be tested. Are the NES sequences that were tested likely to be in long loops or disordered regions of the proteins? Otherwise why would they be expected to be accessible to CRM1? "

This is a simple request to examine the potential NES sequences within the full length protein, to see if the NES is located in a region of the protein which is potentially accessible to CRM1. No bench experiments are necessary. The goal is to see if the potential NES sequences are found in 1) folded domains or 2) in disordered regions. The authors can use many straightforward and user-friendly web tools (PDB, Uniprot, pfam etc.) to see where folded domains are in each of the proteins. If no information is obtained that way, they can also predict folded domains by predicting secondary structure using a tool such as PredictProtein. They can look for disordered regions in each protein using tools such as Dispred. NES sequences that are found within a folded domain are very unlikely to be functional NESs and those in disordered regions are more likely to be accessible and functional.

Author Rebuttal

Rebuttal TRA-18-0758.R1

Dear Dr. Tooze,
please find enclosed the modification as requested by the referees. I hope the changes will meet their expectations.
Best regards
Susanne Bailer

Referee: 1

Comments to the Author
The authors have done a very good job in considering the reviewer's comments on their study. They have taken care of most my comments, and the manuscript has improved considerably. The authors have decided against shortening some redundancies, but they seem to prefer their manuscript to remain like that. I have some few minor comments left.

◊ Thank you again for all the comments that really helped to improve the manuscript.

1. At many (all?) places the tegument protein name pUL37 has been replaced by pUL37d11. I presume this is a spelling error which should be corrected. If not, please provide an explanation; is this a truncated version of pUL37?

◊ Yes indeed this is a truncated version lacking residues 1-11. This had previously been stated in the Materials and methods section (in yellow): „These include the construct UL36a: nucleotides (nct) 1-3000; UL36b: 3001-6000; UL37d11: nct 34-3372; UL46a: nct 1-1008; UL46b: nct 1009-2157 (28).” And is now added to the results section: “All proteins were expressed in full length except for pUL36a (aa 1-1000), pUL36b (aa 1001-2000), UL37d11 (aa 12-1124), UL46a (aa 1-336), and UL46b (aa 337-719).” In addition, we have included this information in Table 2.

2. In the discussion, the authors mention that they only covered experimentally a N-terminal piece of the tegument protein pUL36 but nowhere provide Information how large this piece was. They should add this information to the summarizing table 2.

◊ We have included this information (pUL36a: aa 1-1000; pUL36b: aa 1001-2000) in Table 2.

3. I presume besides pUL36, all other constructs expressed full-length proteins. If not, the domains that have been used in the experiments should be indicated in tables 2 and 3.

◊In addition to pUL36 and pUL37, other proteins were truncated. This had previously been stated in the Materials and methods section: „These include the construct UL36a: nucleotides (nct) 1-3000; UL36b: 3001-6000; UL37d11: nct 34-3372; UL46a: nct 1-1008; UL46b: nct 1009-2157 (28).” And is now added to the results section: “All proteins were expressed in full length except for pUL36a (aa 1-1000), pUL36b (aa 1001-2000), UL37d11 (aa 12-1124), UL46a (aa 1-336), and UL46b (aa 337-719).” In addition, we have included this information in Table 2.

Referee: 2

Comments to the Author
The authors did not address comment #5 of reviewer 2 -
"5. The authors did not analyze the locations of the putative NESs that they mutated in Fig 3B, within their full-length protein sequences. With the exception of the sequences in pUL4, pUL13 and pUL37, the other sequences scored really low and may not be accessible within the full-length proteins. Curiously the highest scoring NES sequence in pUL37 was not picked to be tested. Are the NES sequences that were tested likely to be in long loops or disordered regions of the proteins? Otherwise why would they be expected to be accessible to CRM1? "

This is a simple request to examine the potential NES sequences within the full length protein, to see if the NES is located in a region of the protein which is potentially accessible to CRM1. No bench experiments are necessary. The goal is to see if the potential NES sequences are found in 1) folded domains or 2) in disordered regions. The authors can use many straightforward and user-friendly web tools (PDB, Uniprot, pfam etc.) to see where folded domains are in each of the proteins. If no information is obtained that way, they can also predict folded domains by predicting secondary structure using a tool such as PredictProtein. They can look for disordered regions in each protein using tools such as Disopred. NES sequences that are found within a folded domain are very unlikely to be functional NESs

and those in disordered regions are more likely to be accessible and functional.

◊ As requested by the reviewer, we have applied the web tool PredictProtein to determine whether the NESes are found in folded and/or disordered regions, respectively. For pUL21 and pUL37, a crystal structure is available that was additionally used to determine the secondary structure of the proteins. The results of this analysis is summarized in the Suppl. Table 1 and pointed out in the results section: „Furthermore, based on in silico analysis and structural data, most of the identified NESes are located in disordered and partial folded regions consistent with their accessibility to CRM1/Xpo1 and thus their functionality (Guttler et al., 2010; Suppl. Table 1).“
